# Structural identifiability of parameters of Anand material model

**DOI:** 10.1038/s41598-025-89360-y

**Published:** 2025-02-15

**Authors:** Jaroslav Rojíček, Jakub Cienciala, Martin Fusek

**Affiliations:** Faculty of Mechanical Engineering, VSB - Technical University of Ostrava, 17. listopadu 2172/15, Ostrava-Poruba, 70800 Czech Republic

**Keywords:** Tension test, Finite element method updating, Anand material model, Calibration, Validation, Mechanical engineering, Computational methods

## Abstract

This article explores the structural identifiability of parameters within non-linear physical models, focussing specifically on the Anand material model. The proposed procedure is structured in two key steps: first, a local analysis identifies the most suitable parameters for testing. Subsequently, these selected parameters, along with their admissible value intervals, are evaluated for global structural dependence. Our innovative numerical approach systematically reduces the parameter count by substituting selected parameters with frozen values (constants). The study employs simulations of the finite element method of a simplified tensile test, utilising the Anand material model. The independent parameters identified through this method are then validated against a tensile test dataset. The validation results indicate a high probability that the use of a reduced parameter set yields unique values for the Anand model parameters in the tensile tests, underscoring the efficacy of our approach.

## Introduction

The idea of an identifiability (structural identifiability) was introduced in^1^. Structural identifiability can be described as follows: “Structural identifiability regards the possibility of giving unique values to model unknown parameters form the available observables, assuming perfect experimental data”, citation is taken from^2^. This property is important for material models where individual parameters often represent specific physical behaviours. The requirement for unique values of these parameters is crucial here and can also simplify the identification of their values. For shape-complex components solved by the finite element method (FEM) that use non-linear material models or combined material models, this is very difficult to verify analytically. A numerical approach is an easier alternative. Furthermore, the numerical approach is not limited to material models, but can be applied in other areas as well. In the article, the proposed procedure is applied to the Anand material model in a simple FEM simulation.

Several approaches can be found in the literature. The methods for determining identifiability can be divided into several areas:

- Taylor series approach^3^,
- Sensitivity-based approach^4–6^,
- Monte Carlo based approach^4^,
- Statistical approach (profile likelihood, Bayesian)^4^,
- Direct test^3^ of equality (1),
- etc.

Sources of nonidentifiability are also analysed in^7^. The method used in the article is based on a direct test and is explained in the next Section.

The material model that we will analyse is commonly called the Anand model, as already mentioned. In^8^ the material model for the rate-dependent deformation of metals at high temperatures was first proposed. Anand material model was designed for large elastic-viscoplastic deformations at high temperatures that can be used for the analysis of hot working of metals. In the following work^9^, Anand model has been further developed. The paper also shows the behaviour of the model in the context of selected experiments and describes the procedure for identifying its parameters. The model unifies creep and rate-dependent plastic behaviour by a flow equation and an evolution equation. However, the two parts are usually treated separately; see, for example,^10^. Various experiments can be used to calibrate the material model: tensile tests, creep tests^10^, compression tests^9^, shear tests^11^.

According to^9^, the material parameters of Anand model are determined by the method of non-linear least squares, see, e.g.^10,11^. Nonlinear least-squares solution methods can be found in the literature, e.g.^12–14^. However, other methods can also be used; see, e.g., particle swarm optimisation^15^, neural networks^16^, or the Nelder-Mead simplex method^17^. The identification of material parameters is based on two approaches.

- The first is based on the analytical relationships given by Anand model,
- The second uses the implementation of Anand model into software that uses finite element methods (FEM).

If the problem is solved using one or more FEM simulations, the finite element model updating (FEMU) is used to identify unknown parameters of the problem. It is also used in this article. In the FEMU approach, the FE simulation is repeatedly solved, where, by changing the parameter values, we try to achieve the best possible agreement with the experimental data. This difference is described using the so-called objective function. Therefore, it is simply a task of minimizing the value of the objective function. The determination of the change in these parameters is carried out using gradient algorithms^18^ or non-gradient algorithms^19^.

The influence of experiments on the identifiability has been considered in detail only for indentation tests. In^20^, a simple model of an elastoplastic material was presented whose parameters were identified from the indentation test. In the paper, seven different combinations of material properties that lead to a single force-displacement curve (indentation) are presented. The unique values of the parameters were shown to be inconclusive. In^21^ the influence of the angle value of the indenter was tested. The authors showed that the use of more shapes (angles) can improve the behaviour studied and refine the parameter values. For two indenter shapes, for example, the problem was solved in^22,23^. Materials that show similar behaviour in simple terms are called “mystical materials”, i.e. materials that can show almost identical force-displacement curves even though they have different elastoplastic properties; see, e.g.^24^. It is shown here that even the use of multiple indenter shapes may not provide unambiguous curves.

We could not find similar articles for the Anand material model. On the other hand,^17^ showed that there could be a similar effect. However, the paper only presented initial numerical tests without a more detailed analysis. Different types of experiments are used to identify the parameters of the Anand material model; see, for example, tensile tests^25^, compression tests^9^, shear tests^11^, and creep tests^26^. The use of indentation tests in combination with the Anand model is not common; it was used in^17^. Vicoplastic material with indentation was used in^27^ but with a different material model. Tensile/compression and creep tests are most commonly used in conjunction with the Anand model. Therefore, tensile tests were chosen for the simulations in this paper. In^28^, the Ohno-Wang and Armstrong-Fedrick models with the non-linear kinematic hardening rule were compared with the Anand model for monotonic and cyclic loading. The Anand model showed significantly worse results. A larger number of material models (including the Anand model) were compared in^29^ with similar results. Therefore, cyclic loading is not included in this analysis.

Anand constitutive models for lead-free solder are very popular. Parameter calibration is performed from a set of experiments, usually tension tests. The resulting values of the parameters for Anand model vary widely; see, for example,^11^. The article presents parameter values for the materials (Sn95.5Ag3.8Cu0.7, SAC387) published by different authors. For the material, the value of one of the parameters is in the range 15.773 - 1.63E+6. Similar data can be found for materials (Sn96.5Ag3.0Cu0.5, SAC305) or (Sn96.5Ag3.5), etc. Different values can be expected, for example, due to the effect of different production technologies of the sample. However, if we compare, for example, the stress-strain curves at different temperatures and strain rates, their shape is similar, and most of the curves lie between the values of 10-50 MPa. This may indicate that the different parameter values are not due to different material behaviour. This problem is also mentioned, for example, in^5^. The problem of non-uniqueness of the material parameters identified during the calibration of the material model is also presented in^30^. This problem is usually referred to as structural identifiability and also arises in the context of experiments. The comparison of two materials is then only possible on the basis of the curves and not on the basis of the parameter values of Anand model. Then we cannot speak of the physical meaning of a material parameter.

For a number of material models, we can get a good idea of the behaviour of the corresponding material based only on the value of the parameter. This feature greatly simplifies the possibility of using the given material model in practice. The identification of parameters itself is becoming relatively easy. Calibration itself is simplified by the increasing computing power of computers, the availability of various applications in commercial software, and the possibilities of so-called artificial intelligence. The uniqueness of parameters and their associated identifiability is therefore very important for material models.

The article is structured as follows. Section 2 presented the basic methods. Section 3 shows an application of the methods to tension tests. The main part of this section is analysis of local and global dependences. Section 4 validates the results on a real set of tension tests. The last key part of the paper is a discussion in Section 5. This describes several points that were identified during the course of the work on this paper. These are related to the current results, but also to possible future directions for the next work.

## Methods

In this Section, we describe ideas that lead to a numerical approach to identify the dependent parameters, called structural identifiability. The structural identifiability can be described by equality (see, for example,^4,31^):

1 $$\begin{aligned} \Gamma ({\textbf {P}}) = \Gamma ({\textbf {P}}_*) \Rightarrow {\textbf {P}} = {\textbf {P}}_*, \end{aligned}$$

where $\Gamma$ represents a model and ${\textbf {P}}$, ${\textbf {P}}_*$ represent sets of parameter values. Practical identifiability is also distinguished, where real data that contain noise is considered. The procedure was divided into two steps:

- Parameter selection using (so-called) the structurally local dependence of the parameters.
- Testing the parameter on (so-called) the structurally global dependence of the parameter. The parameter values are bounded by an interval, and the solution is performed at the chosen level of precision.

This Section is structured as follows; first, the notation and terminology are described (Section 2.1), then there are defined objective functions (Section 2.2) and the Anand material model (Section 2.3). The local and global structural dependence of the parameters is defined in Section 2.4). The basic procedure for determining the so-called structural identifiability of the parameters is presented in Section 2.5. In the last Section 2.6 we describe the FEMU approach.

### Notations and terminology

In this paper, we use the following notation: $\mathbb {N}^+$ is the set of positive whole numbers, $\mathbb {R}$ is the set of all real numbers, and $\mathbb {R}^+$ is the set of positive real numbers without zero. A relation $\Gamma$ maps the inputs ${\textbf {P}}$ and ${\textbf {X}}$ to an output ${\textbf {Y}}$, as follows:

2 $$\begin{aligned} {\textbf {Y}} = \Gamma ({\textbf {P}},{\textbf {X}}). \end{aligned}$$

In this paper, we will simulate mechanical experiments using the Finite Element Method (FEM). Each experiment can be described by the shape of the sample (for example, the diameter and length of the simulated part of the sample), the set of test conditions (e.g., temperature, strain rate), and an output data set. The data set typically includes three types of values: the value representing the actual time, the loading state for the actual time, and the measured data for the actual time. Let ${\textbf {Y}}$ have the same structure, and let ${\textbf {Y}}$ be called an output data set. A more detailed description of the individual parts of (2) follows:

- $\Gamma ()$ represent a numerical simulation (FEM) of an experiment/experiments.
- ${\textbf {P}}=\{ p_i: i = 1, 2, \ldots , N \}$ is a vector of parameters, $p_i \in \mathbb {R}$ is a parameter, and $N \in \mathbb {N}^+$ is the number of parameters in the set. For some material models, the value of a parameter can be constrained, for example, $\exists p_i \in {\textbf {P}}: p_i>0$. Let is ${\textbf {P}} \in \mathbb {P}$, where $\mathbb {P}$ is a set of all possible ${\textbf {P}}$. In our case, the parameters refer exclusively to Anand material model.
- The superscript ${\textbf {P}}^i$ denotes a parameter of interest. We will use the following for the parameter set ${\textbf {P}}^i = \{ p_1, \ldots , p_i + \epsilon _i, \ldots , p_N \}: \epsilon _i \in \mathbb {R}^+$, and ${\textbf {P}}^{\lnot i} = \{ p_1 + \epsilon _1, p_2 + \epsilon _2, \ldots , p_i,\ldots , p_N + \epsilon _N\}:\epsilon _1,\epsilon _2, \ldots \in \mathbb {R}$.
- The subscripts ${\textbf {P}}_{i,j}$ denote solution cycles. Here, *i* is a solution variant (variant of the initial values) and *j* is an actual cycle value. In short, ${\textbf {P}}_*$ denotes a variant of the vector of parameters.
- We will use only tension tests. The loading states are defined by displacements. ${\textbf {X}}= \{{\textbf {t}}, {\textbf {u}}\}$ is a set of independent variables (input), and ${\textbf {t}}, {\textbf {u}}$ are the time and displacement vectors, respectively. In the context of a numerical simulation or a simulated experiment, it can be interpreted as a loading state.
- Let us have a set of experiments $\Psi = \{ \psi _i: i = 1, 2, \ldots , N_{\Psi } \}$, where $\psi _i$ denotes the *i*-th experiment and $N_{\Psi }$ is the number of experiments in the set.
- Basic vectors ${\textbf {t}}, {\textbf {F}}, {\textbf {u}}$ are defined as follows:
  3 $$\begin{aligned} \begin{aligned} {\textbf {t}}^{\psi }&= [ t_j^{\psi }:j = 1, 2, \ldots , N^{\psi }_T]\ (\text {time points}), \\ {\textbf {F}}_i^{\psi }&= [ F_{i,j}^{\psi }:j = 1, 2, \ldots , N^{\psi }_T] \ (\text {forces}), \\ {\textbf {u}}_i^{\psi }&= [ u_{i,j}^{\psi }:j = 1, 2, \ldots , N^{\psi }_T] \ (\text {displacements}). \end{aligned} \end{aligned}$$
  Here, ${\textbf {F}}$ is the vector of force, $N^{\psi }_T$ denotes the number of time points. The meaning of the upper and lower index is similar to that of the set of parameters ${\textbf {P}}$. The superscript $\psi$ denotes an experiment, the subscript *i* is an actual cycle value, and *j* represents a position in a vector. For simplicity, we assume that the number of points and its position in time for vectors ${\textbf {F}}_i^{\psi }$, ${\textbf {u}}_i^{\psi }$ are the same.
- ${\textbf {Y}}$ is the output data set, as mentioned above. The structure of the data set can be symbolically described as ${\textbf {Y}}= \{{\textbf {t}}, {\textbf {F}}, {\textbf {u}} \}$ and has the same structure for all simulated experiments. Therefore, ${\textbf {Y}}$ (an output data) always contains ${\textbf {X}}$ (a loading set) as well.
- The meaning of the upper and lower indexes of ${\textbf {Y}}$ is similar to ${\textbf {P}}$, ${\textbf {t}}, {\textbf {F}}, {\textbf {u}}$. The superscript will indicate an experiment $\psi \in \Psi$. If the value of the subscript is $i = 0$, then the output data represent the data from the experiment $\psi$. If the value of the subscript is $i>0$, then it is data from the *i*-th simulation of the experiment $\psi$.
- We will also refer to ${\textbf {X}}^{\psi }$ as the loading state for experiment $\psi$ and $\Gamma ^{\psi } ()$ as the simulation model for experiment ${\psi }$.
- The tensile load prevails in the experiments used; therefore, we will indicate the values in the direction of the sample axis without a direction index (displacement *u*, force *F*, strain $\varepsilon$ and stress $\sigma$). If we use other quantities, for example equivalent stress, it will be highlighted and marked differently.

### Objective function

An objective function indicates the agreement of the two sets of data ${\textbf {Y}}^{\psi }_i$ and ${\textbf {Y}}^{\psi }_j$ for the experiment ${\psi }$. This type of objective function will be called a partial objective function (POF)^32^. It will be denoted as follows:

4 $$\begin{aligned} \Vert {\textbf {Y}}_i,{\textbf {Y}}_j \Vert ^{\psi }, \end{aligned}$$

where $\Vert {\textbf {Y}}_i,{\textbf {Y}}_j \Vert ^{\psi }$ is a norm for POF calculation. The norm $\Vert {\textbf {Y}}_i,{\textbf {Y}}_j \Vert ^{\psi }$ for an experiment ${\psi } \in \Psi$ is calculated as:

5 $$\begin{aligned} \Vert {\textbf {Y}}_i,{\textbf {Y}}_j \Vert ^{\psi }= \sqrt{ \frac{ \sum _{k=1}^{N_T^{\psi }} (F_{i,k}^{\psi } - F_{j,k}^{\psi })^2}{\sum _{k=1}^{N_T^{\psi }} ( F_{i,k}^{\psi } )^2}}. \end{aligned}$$

The second type of objective function for the set of experiments $\Psi$ will be called a total objective function (TOF)^32^. It will be denoted as:

6 $$\begin{aligned} \Vert {\textbf {Y}}_i,{\textbf {Y}}_j \Vert ^\Psi = \Vert {\textbf {Y}}^{\psi }_i, {\textbf {Y}}^{\psi }_j: \psi = 1, 2, \ldots , N_{\Psi } \Vert . \end{aligned}$$

And the norm $\Vert {\textbf {Y}}_i,{\textbf {Y}}_j \Vert ^\Psi$ for a set of experiments $\Psi$ is calculated as

7 $$\begin{aligned} \Vert {\textbf {Y}}_i,{\textbf {Y}}_j \Vert ^\Psi = \sqrt{\frac{ \sum _{\psi =1}^{N_{\Psi }} (\Vert {\textbf {Y}}_i,{\textbf {Y}}_j \Vert ^{\psi })^2}{N_{\Psi }}}. \end{aligned}$$

Note: Comparison of source data (for example, experimental data) and data from the i-th simulation is marked as $\Vert {\textbf {Y}}_0,{\textbf {Y}}_i \Vert ^{\psi }$, or $\Vert {\textbf {Y}}_0,{\textbf {Y}}_i \Vert ^{\Psi }$.

### Anand material model

Anand material model was designed for the rate-dependent deformation of metals at high temperature, and is used for the creep and rate-dependent plastic behavior as was presented in introduction section. For one dimension, the stress equation can be described as:

8 $$\begin{aligned} \sigma = c s. \end{aligned}$$

Where *c* is a function of a strain rate and temperature with constraint $c<1$, and *s* is an internal variable which represent deformation resistance. *c* is calculated as follows

9 $$\begin{aligned} c = \frac{1}{\xi } sinh^{-1} \left[ \left( \frac{\dot{\varepsilon }_p}{A} exp\left( \frac{Q}{RT} \right) \right) ^m \right] , \end{aligned}$$

where $\xi$ is a stress multiplier, $\dot{\varepsilon }_p$ is the inelastic strain rate, *A* is a material constant, *Q* is the activation energy, *R* is the universal gas constant, *T* is a absolute temperature, and *m* is a strain sensitivity exponent. The second part includes the evolution equation as follows.

10 $$\begin{aligned} \dot{s} = h_0 \left| 1-\frac{s}{s^*} \right| ^a sign\left( 1-\frac{s}{s^*} \right) \dot{\varepsilon }_p, \ s^* = \hat{s} \left( \frac{\dot{\varepsilon }_p}{A} exp\left( \frac{Q}{RT} \right) \right) ^n . \end{aligned}$$

Where $h_0$ is a deformation hardening-softening constant, *a* is a strain sensitivity exponent, $s^*$ is a saturation value, $\hat{s}$ is a deformation resistance coefficient, and *n* is a rate sensitivity exponent. If $s<s^*$ then $sign\left( 1-\frac{s}{s^*} \right) = 1$ and the first equation of (10) can be modified to

11 $$\begin{aligned} ds = h_0 \left| 1-\frac{s}{s^*} \right| ^a d\varepsilon _p, \end{aligned}$$

and integrated to

12 $$\begin{aligned} s = s^* - \left[ (s^* - s_0)^{1-a} + \varepsilon _p h_0 (a-1) (s^*)^{-a} )\right] ^{\frac{1}{1-a}} , \end{aligned}$$

where $s_0$ is an initial value of the internal variable *s* (deformation resistance).

The presented Anand model contains nine parameters: $\xi$, *A*, *Q*/*R*, *m*, $h_0$, *a*, $s_0$, $\hat{s}$, and *n*. We consider the material parameters describing the behaviour in the linear elastic region (Young modulus^33^, Poisson’s number^34^) to be known.

There are modifications of the Anand model. For example, in^35^ modifications were tested that the parameter $h_0$ is a function of a temperature *T* and a plastic strain rate $\dot{\varepsilon }_p^2$ as follows:

13 $$\begin{aligned} h_0 = A_0 + A_1 T + A_2 T^2 + A_3 \dot{\varepsilon }_p + A_4 \dot{\varepsilon }_p^2. \end{aligned}$$

In^36^ a modification was tested which additionally adds the parameter $s_0$ as a function of a temperature *T*:

14 $$\begin{aligned} s_0 = S_0 + S_1 T + S_2 T^2. \end{aligned}$$

Both modifications are implemented in ABAQUS and contain fifteen parameters^36^. Different modifications of the parameter $h_0$ were used in^37^ as follows:

15 $$\begin{aligned} h_0 =a_h \left( \frac{\dot{\epsilon }_p}{A} \right) ^{n_1} \left[ exp\left( \frac{Q}{RT} \right) \right] ^{n_2}, \end{aligned}$$

where $a_h$, $n_1$, and $n_2$ are new parameters. In^38^ an idea was tested that all Anand parameters as linear temperature dependent, the model contains 18 parameters.

### Structural identifiability

The methods for estimating structural identifiability were presented in the introductory section; in this paper the direct method^3^ is used. The number of experiments is limited only by the available data or computer power, and FEM is used to simulate even complex experiments. Therefore, the FEMU approach is used for the numerical solution of the identifiability of parameters.

At first we define the agreement of two possible sets of output data ${\textbf {Y}}^{\Psi }$, ${\textbf {Y}}^{\Psi }_*$ on a set of experiments $\Psi$ as follows

#### Definition 1

Let ${\textbf {Y}}^{\Psi }_*$ lies in a $\epsilon$-neighbourhood of ${\textbf {Y}}^{\Psi }$ as follows

16 $$\begin{aligned} {\textbf {Y}}^{\Psi }_* \in N_{{\textbf {Y}}}^\epsilon ({\textbf {Y}}^{\Psi }), \end{aligned}$$

when

17 $$\begin{aligned} \Vert {\textbf {Y}},{\textbf {Y}}_* \Vert ^\Psi \le \epsilon , \end{aligned}$$

where $\epsilon \in \mathbb {R}^+$ represents a level of agreement between two sets of data ${\textbf {Y}}^{\Psi }$, ${\textbf {Y}}_*^{\Psi }$.

A neighborhood of parameter $p_i$ can be defined as follows.

#### Definition 2

Let ${\textbf {P}}_*$ lies in a $\epsilon _i$-neighbourhood of ${\textbf {P}}$ as follows

18 $$\begin{aligned} {\textbf {P}}_* \in N_{{\textbf {P}}}^{\epsilon _i,i}({\textbf {P}}), \end{aligned}$$

when

19 $$\begin{aligned} {\textbf {P}}_* = \{p_1, \ldots , p_{i-1}, p_i^*, p_{i+1}, \ldots , p_{N} \}, \end{aligned}$$

where $$p_i< p_i^* \le p_i + \epsilon _i$$, and $$\epsilon _i \in \mathbb {R}^+$$.

Notes:

- $${\textbf {P}} = \{p_1, \ldots , p_{i-1}, p_i, p_{i+1}, \ldots , p_{N} \} : p_i \not < p_i \le p_i + \epsilon _i \Rightarrow {\textbf {P}} \not \in N_{{\textbf {P}}}^{\epsilon _i,i}({\textbf {P}})$$.
- Let s $${\textbf {P}}_* \in N_{{\textbf {P}}}^{\epsilon _i,i}({\textbf {P}})$$ then $$(\epsilon _i > 0) \Rightarrow ({\textbf {P}}_* \ne {\textbf {P}} )$$.
- We also assume $\forall {\textbf {P}}_* \in N_{{\textbf {P}}}^{\epsilon _i,i}({\textbf {P}}): {\textbf {P}}_*\in \mathbb {P}$.
- $${\textbf {P}}^i = \{p_1, \ldots , p_{i-1}, p_i+\epsilon _i , p_{i+1}, \ldots , p_{N} \} : p_i< p_i + \epsilon _i \le p_i + \epsilon _i \Rightarrow {\textbf {P}}^i \in N_{{\textbf {P}}}^{\epsilon _i,i}({\textbf {P}})$$.

#### Local analysis

The goal is to determine the appropriate order of parameters for their subsequent tests. Neighborhood definitions can be used to define a characteristic of a structurally local dependence $\lambda _{i,j}$. Now, let us summarize our considerations:

- Let’s have a constant $\epsilon _i \in \mathbb {R}^+$ then ${\textbf {P}}^i \in N_{{\textbf {P}}}^{\epsilon _i,i}({\textbf {P}})$, and from (2) ${\textbf {Y}}^{\Psi }_i = \Gamma ^{\Psi }({\textbf {P}}^i, {\textbf {X}}^{\Psi })$.
- And let us also have a variable $\epsilon _j \in \mathbb {R}$: ${\textbf {P}}^j \in N_{{\textbf {P}}}^{\epsilon _j,j}({\textbf {P}})$, ${\textbf {Y}}^{\Psi }_j = \Gamma ^{\Psi }({\textbf {P}}^j, {\textbf {X}}^{\Psi })$.
- Then the similarity of the behavior of $p_i$ and $p_j$ around the point ${\textbf {P}}$ can be expressed as a value of a structurally local dependence $\lambda _{i,j}$ and is defined as follows:

##### Definition 3

A value of the **structurally local dependence** $\lambda _{i,j}$ between the parameters $p_i, p_j \in {\textbf {P}}$ for a given $\varepsilon _i \in \mathbb {R}^+$ is a minimum value of $\Vert {\textbf {Y}}_i,{\textbf {Y}}_j \Vert ^\Psi$.

20 $$\begin{aligned} \lambda _{i,j} = min_{\varepsilon _i} \Vert {\textbf {Y}}_i,{\textbf {Y}}_j \Vert ^\Psi . \end{aligned}$$

Notes:

- Put simply, $\lambda _{i,j}$ tells, how difficult is to replace changes in the value of the parameter *i* by changes in the value of the parameter *j*.
- The value of $\lambda _{i,j}$ is influenced by the values of the vector ${\textbf {P}}$ and the value of $\varepsilon _i$.
- The value of $\varepsilon _i$ must be small enough compared to the value of the parameter $p_i$. Values in the range of $\varepsilon _i = (0.1 \cdots 0.01) p_i$ were tested. At higher values, there were problems with the convergence of some simulations, or a parameter value ($p_j$) went outside the allowed range.
- It is obvious that for $i = j \ \Rightarrow \ min_{\varepsilon _i} \Vert {\textbf {Y}}_i,{\textbf {Y}}_j \Vert ^\Psi = 0$.

The values of local dependence $\lambda _{i,j}$ can be arranged in a matrix $\Lambda$ (dimensions NxN) and contains zeros on the diagonal as follows

##### Definition 4

A local dependence matrix $\Lambda$ is calculated as

21 $$\begin{aligned} \Lambda = \begin{bmatrix} 0 & \lambda _{1,2} & \lambda _{1,3}& \lambda _{1,4}& & ...& & \lambda _{1,N} \\ & 0 & \lambda _{2,3}& \lambda _{2,4}& & ... & & \lambda _{2,N} \\ & & & & ... & \\ & & & & & 0& \lambda _{N-1,N} & \lambda _{N-2,N} \\ & SYM & & & & & 0 & \lambda _{N-1,N} \\ & & & & & & & 0 \\ \end{bmatrix}. \end{aligned}$$

Note: For simplicity, we assume that the matrix is symmetric ($\lambda _{i,j}= \lambda _{j,i}$). This would only be true if the values of $\varepsilon _i$ and $\varepsilon _j$ are interchanged: If $\varepsilon _j = argmin_{\varepsilon _i} \Vert {\textbf {Y}}_i,{\textbf {Y}}_j \Vert ^\Psi$ then $min_{\varepsilon _i} \Vert {\textbf {Y}}_i,{\textbf {Y}}_j \Vert ^\Psi = min_{\varepsilon _j} \Vert {\textbf {Y}}_i,{\textbf {Y}}_j \Vert ^\Psi$.

The sum of all the values in the row *i* of the matrix gives us a cumulative value:

##### Definition 5

A local dependence coefficient $\Lambda _i$ for the *i* th parameter is calculated as

22 $$\begin{aligned} \Lambda _i = \sum _{j = 1}^N \lambda _{i,j} \end{aligned}$$

This allows us to estimate the local dependence of one parameter (*i*) on all others and it serves to select parameters for testing global dependence. We assume that the lowest value of $\Lambda _i$ means that the parameter (*i*) is most easily replaced by the other parameters.

Note: This step could be solved using a classical approach like non-linear regression. Testing two parameters is much simpler and faster, that is why it was used.

#### Global analysis

The goal is to show that the selected parameter is dependent; for this the negation of the relationship (1) is used. A global dependency of a parameter $p_i$ can be defined in a similar way, except that the value of the parameter is not limited by a neighborhood. Therefore, changing the value of the *i* -th parameter (known as ${\textbf {P}}^i$) will be replaced by changing the values of the other parameters (known as ${\textbf {P}}^{\lnot i}$ ) for the entire interval of the *i* -th parameter. The basic consideration can be summarized in several points:

- Let’s have a constant $\epsilon _i \in \mathbb {R}^+$ then ${\textbf {P}}^i \in N_{{\textbf {P}}}^{\epsilon _i,i}({\textbf {P}})$, and ${\textbf {Y}}^{\Psi }_i = \Gamma ^{\Psi }({\textbf {P}}^i, {\textbf {X}}^{\Psi })$.
- We are looking for ${\textbf {P}}^{\lnot i} = \{ p_1 + \epsilon _1, p_2 + \epsilon _2, \ldots , p_i,\ldots , p_N + \epsilon _N\}: {\textbf {P}}^{\lnot i} \in \mathbb {P}$, where $\epsilon _1 \in \mathbb {R}$,$\epsilon _2 \in \mathbb {R}$,... $\epsilon _N \in \mathbb {R}$, ${\textbf {Y}}^{\Psi }_{\lnot i} = \Gamma ^{\Psi }({\textbf {P}}^{\lnot i}, {\textbf {X}}^{\Psi })$.
- Then the similarity of the behavior of ${\textbf {P}}^i$ and ${\textbf {P}}^{\lnot i}$ can be expressed as $min_{\epsilon _i}\Vert {\textbf {Y}}_i,{\textbf {Y}}_{\lnot i} \Vert ^\Psi$.
- Which we can briefly describe in the following definition:

##### Definition 6

Parameter $p_i \in {\textbf {P}}$ is a ** structurally globally dependent** with precision $\epsilon \in \mathbb {R}^+$, if for

23 $$\begin{aligned} \forall {\textbf {P}}^i \in \mathbb {P} \ \text {exist} \ {\textbf {P}}^{\lnot i}\in \mathbb {P} \ \text {such that} \ \Vert {\textbf {Y}}_i,{\textbf {Y}}_{\lnot i} \Vert ^\Psi < \epsilon , \end{aligned}$$

where $$\epsilon _i \in \mathbb {R}^+$$, $$\epsilon _1, \epsilon _2, \ldots , \epsilon _N \in \mathbb {R}$$.

Notes:

- Put simply, we will try to satisfy the negation of (1) numerically.
- The parameter $p_i$ is tested for 10 values of the selected interval. The size of the interval is determined from data published in the literature.
- We assume that for ${\textbf {P}}_0, {\textbf {P}}^i \in \mathbb {P}$ is met: if $\epsilon _i \not = 0$ then $\Vert {\textbf {Y}}_0,{\textbf {Y}}_i \Vert ^\Psi > 0$. Therefore, with respect to the numerical solution, the value of $\epsilon _i$ must be large enough.
- Regarding the numerical solution, it is also necessary to take into account the possibility that ${\textbf {P}}^{\lnot i}$ will not be found by the chosen method, although it exists.

### Solution procedure

The solution procedure consists of two different parts, as was presented in the previous section. First, the local structural dependence matrix is determined. On the basis of its evaluation, a parameter is selected for global dependency testing. The procedure can be summarized in several points:

1. Start: all parameters are considered independent.
2. A local structural dependency matrix $\Lambda$ ((20) and (21)) is calculated.
3. For each independent parameter, the value of the local dependence coefficient $\Lambda _i$ (22) is calculated.
4. The parameter $p_i$ with the lowest value of $\Lambda _i$ is selected.
5. The selected parameter $p_i$ is tested for global structural dependence (Definition 6) for 10 values of the possible interval of $p_i$.
6. If the parameter $p_i$ is dependent, then it is removed from a list of independent parameters. The row and column corresponding to it are removed from the local structural dependence matrix $\Lambda$, and the procedure continues with Step 3.
7. Finish: Otherwise,the parameter $p_i$ is independent. The solution procedure is terminated.

The Finite Element Method Updating approach (FEMU) was used to determine the values of the local structural dependency matrix $\Lambda$ and to test global dependency.

### FEMU method

The FEMU method is used to determine the parameter values for the minimum of TOF. TOF may include one or more finite element models, as presented in Section 2.2. Calculations of the objective function value (and therefore the FEM) take place in a cycle, and the parameter values change in individual cycles so that the objective function value decreases. The methods used to update the parameters can be divided into these basic parts.

1. Gradient methods, the structure of the task is used in the solution.
2. Non-gradient methods, the solution can be based on statistical, or evolutionary approaches, etc. In the paper, we use the so-called Nelder - Mead (Simplex) algorithm.

The FEMU method can be briefly described as follows:

24 $$\begin{aligned} {\textbf {P}}_* = FEMU ({\textbf {P}}_{1}, {\textbf {Y}}^{\Psi }_0, \Gamma ^{\Psi },\text {N}_F, \epsilon , \text {ANMS}, \cdots ). \end{aligned}$$

Where ${\textbf {P}}_*$ is a vector of the values of the resulting parameters, ${\textbf {P}}_{1}$ is a vector of the values of the initial parameters, ${\textbf {Y}}^{\Psi }_0$ is a set of data (loading states and experimental data), $\Gamma ^{\Psi }$ represents a set of simulation models, $\text {N}_F$ is the maximum number of TOF calculations, $\epsilon$ is the required TOF value; see (23). ANMS is an abbreviation for the optimization method used, for example, the adaptive Nelder-Mead Simplex algorithm (ANMS). There can also be parameters for the setting of the method.

In the paper, two optimization methods are used.

- The first is for the identification of the value of the local dependence $\lambda _{i,j}$, where only one value is identified. Since the interval of allowed values is known for each parameter, many methods can be used. In this paper, we use Newton’s method. The method is known, so it is not described here. The following termination criterion was used: $\mid \Vert {\textbf {Y}}_0,{\textbf {Y}}_{k} \Vert ^\Psi - \Vert {\textbf {Y}}_0,{\textbf {Y}}_{k-1} \Vert ^\Psi \mid < \epsilon _L$, where *k* is the current value of the number of cycles and $\epsilon _L$ is the required precision.
- The second is for the identification of the global dependence, where the number of search parameters that can be changed is larger. We use the Nelder-Mead simplex method^17^ with the modification described in^39^ to solve. The method is known, so it is not described here. The termination criterion derived from the Nelder-Mead algorithm $(w-b)/b<\epsilon _G$ was used, where *w* is the worst TOF value in the simplex, *b* is the best value in the simplex, and $\epsilon _G$ is the precision required.

Note: Since the set of experiments $\Psi$ contains tens of experiments, the simulations are run in parallel to reduce computational time.

## Application

This section presents an application of the procedure described in the previous section. Due to the large number of calculations, a calculation model with one axisymmetric element and FEM was chosen as the method of simulating the experiments. Furthermore, a tensile test was chosen at different temperatures and loading rates, which is among the typical types of experiments for the calibration of Anand model parameters. These simplifications may affect the results; however, in our opinion, they are sufficient for testing the proposed approach.

This section is structured as follows: Section 3.1 describes the reduced axisymmetric model used for all simulations in this section. The intervals in which the parameters can move are described in Section 3.2. In Section 3.3 there is an explanation of the method of obtaining source data (fictitious data set). In Section 3.4 the minimum value of the objective function is determined, for which we assume that the simulations are identical to the experiments. In Section 3.5, the analysis of local dependence is described, on the basis of which subsequently selected parameters are tested. In Section 3.6, these selected parameters are gradually tested for global structural dependence.

### Reduced axisymetric model

The number of experiments can be reduced by using samples with a more complex shape; see, e.g.,^40,41^ where data for multiple cross sections can be obtained using DIC. This increases the amount of data obtained from a single test and thus the resulting quality of the parameters for the identified material model. The strain values in the direction of the sample axis $\varepsilon _y$ obtained at the narrowest point in the cross section of the sample then correspond to the loadings of one element.

**Figure 1:**
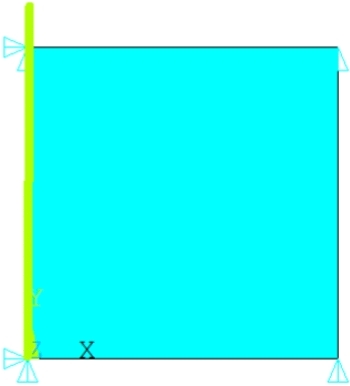
Mesh for axisymetric simulation model of the tension test.

This model is basic for testing the parameters of structural dependence (Section 2.4). It is used in many calculations; therefore, a reduced model was chosen that includes only one element. The model is reduced to one axisymmetric element (Plane 182, axisymmetric setting) with dimensions of 1x1mm. The asymmetric axis is the *y* axis, vertical in Figure 1), where the model is shown.

On the basis of^40^, non-linear loading was used. The selected points of the nonlinear curve (“Nonlinear”) are in Table 1.

**Table 1:** Points of the loading curve.

i [-]	1	2	3	4	5	6	7	8	9
Time [s]	0	112	225	393	647	1026	1589	2152	2714
Disp. [mm]	0	0.0002	0.00033	0.0004	0.00053	0.00061	0.00115	0.00222	0.00388

i [-]	10	11	12	13	14	15	16	17	18
Time [s]	3277	3840	4402	4965	5528	6090	6653	7215	7778
Disp. [mm]	0.00619	0.00914	0.01224	0.016	0.02019	0.0249	0.03	0.0355	0.0412

i [-]	19	20	21	22	23	24	25		
Time [s]	8341	8903	9466	10029	10591	10922	11253		
Disp. [mm]	0.0474	0.0538	0.0605	0.0679	0.0753	0.0798	0.0843		

The lower side of the model is fixed and the upper side is loaded by a gradually increasing displacement (see Table 1). Five different temperatures (20, 40, 60, 80, 100 $^{\circ }$C) and five different strain rates (multiply time coefficient 1, 0.1, 0.01, 0.001, 0.0001) are solved. So, a total of 25 fictitious experiments.

### Interval selection

The basic determination of the interval of values for the individual parameters of Anand material model is based on the used software (Ansys), in which the following limits were identified (basic intervals):

- Condition: If $s_0 \le 0$, then $s_0 = 0.01$, ($p_0 \in [0.01,\infty )$,
- Condition: If $A \le 0$, then $A = 0.01$, ($p_2 \in [0.01,\infty )$,
- Condition: If $m \le 0$, then $m = 0.01$, ($p_4 \in [0.01,\infty )$,
- Condition: If $h_0 \le 0$,then $h_0 = 0.01$, ($p_5 \in [0.01,\infty )$),
- Condition: If $n \le 0$, then $n = 0.0000001$, ($p_7 \in [0.0000001,\infty )$),
- Condition: If $a \le 1$, then $a = 1.00001$, ($p_8 \in [1.00001,\infty )$).

Note: when the condition was met, a solver error occurred; therefore, in this case the parameter values were replaced by another value near the found limit, and the calculation continued to identify with this value.

The intervals are not limited from above, so it would not be possible to test the entire interval numerically (according to (23)). Therefore, the intervals were reduced by the values given for similar materials^11^. The resulting global intervals are as follows:

- $$s_0 = p_0 \in [0.01,50]$$ [MPa] ,
- $$Q/R = p_1 \in [6000,15000]$$ [K],
- $$A = p_2 \in [10,20000]$$ [1/s],
- $$\xi = p_3 \in [1,10]$$ [-],
- $$m = p_4 \in [0.1,4]$$ [-],
- $$h_0 = p_5 \in [100,50000]$$ [MPa],
- $$\hat{s} = p_6 \in [3,100]$$ [MPa],
- $$n = p_7 \in [0.005,0.04]$$ [-],
- $$a = p_8 \in [1.00001,2]$$ [-].

These global intervals were used to test the global dependence of the parameter. Therefore, it only applies to one tested parameter; the basic intervals apply to the other parameters.

### Data sources

The identification of material parameters is usually based on experiments. This article focuses primarily on the behavior of the material model; therefore, the following constraints were chosen:

1. Assume that the behavior of a fictive material used (for example SAC305) can be described very well by the Anand model (^11,26^, etc.).
2. Identifiability will be addressed only for tensile experiments^42^.
3. We assume that we have a number of experiments ($\Psi , {\textbf {Y}}_0^\Psi$) at our disposal.
4. From these experiments, it was possible to identify the parameter values ${\textbf {P}}_*$ of Anand model with sufficient precision.
5. If we use the Anand model correctly and know the values of its material parameters ${\textbf {P}}_*$, then we can assume $\Vert {\textbf {Y}}_0,{\textbf {Y}}_* \Vert ^\Psi \approx 0$. For the purposes of this article, we assume that $\Vert {\textbf {Y}}_0,{\textbf {Y}}_* \Vert ^\Psi = 0$ is true and we know the values of the parameters ${\textbf {P}}_* = {\textbf {P}}_0$.
6. Parameters are called fictive parameters ${\textbf {P}}_0$. The corresponding data are called fictive data ${\textbf {Y}}_0^\Psi$ from fictive experiments $\Psi$. Parameters ${\textbf {P}}_0$ can be, for example, as shown in Table 2.
7. From the previous points it follows that for any $\epsilon$ value there exists at least one vector of parameters ${\textbf {P}}_* = {\textbf {P}}_0$, so that $\Vert {\textbf {Y}}_{0},{\textbf {Y}}_* \Vert ^\Psi = 0 < \epsilon$. The condition only applies to fictitious experiments, as real experiments may be burdened by, for example, measurement error. Data from real experiments are used in the validation part.

**Table 2:** Values of the parameters ${\textbf {P}}_0$ for a fictive material.

$s_0$ [*MPa*]	*Q*/*R* [*K*]	*A* [1/*s*]	$\xi$ $[-]$	*m* $[-]$	$h_0$ [*MPa*]	$\hat{s}$ [*MPa*]	*n* $[-]$	*a* $[-]$
21.398	10494.2	6103.5	4.0	0.2	6139.5	26.4	0.01	1.9075

In this way, we can obtain fictitious data from any test that can be simulated using FEM.

Note: fictitious data represent only the behavior of the material model, not the behavior of the real material.

### Determining the accuracy of the simulation

**Figure 2:**
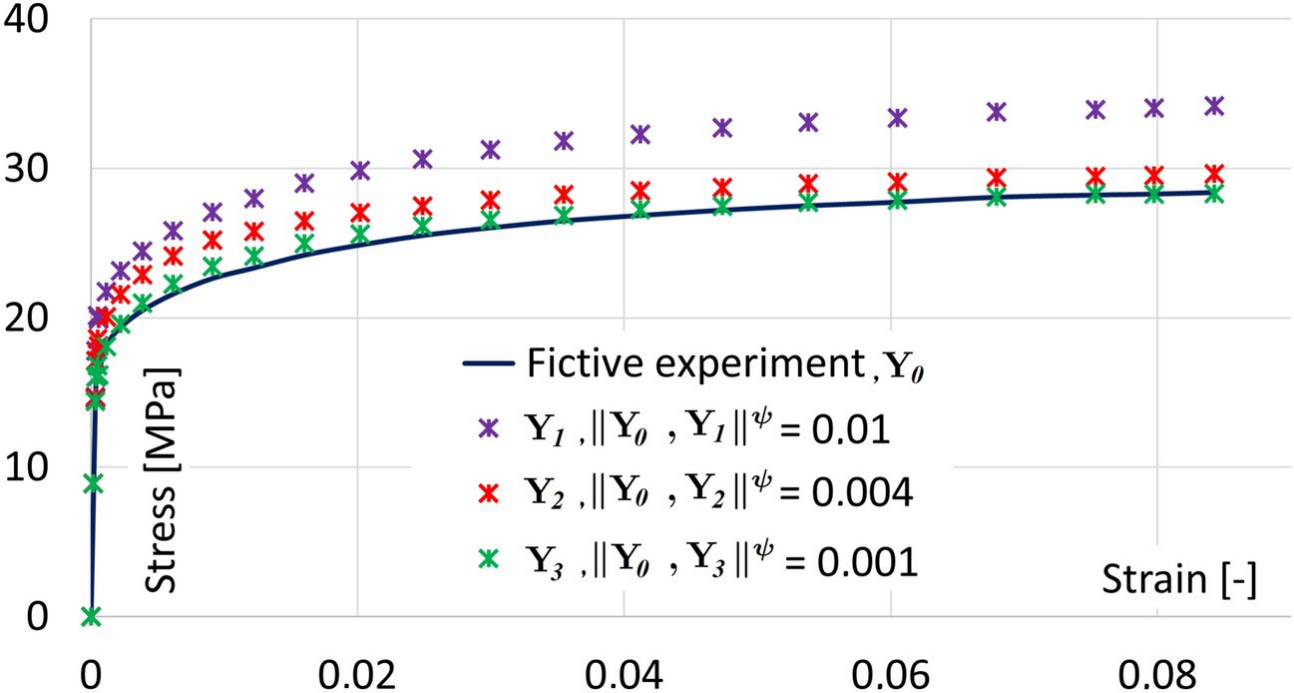
Comparison the curves and values of the norm $\Vert {\textbf {Y}}_{0},{\textbf {Y}}_* \Vert ^{\psi }, \ * = 1, 2, 3,$ in Stress-Strain graph.

In this paper, we use the value of norm $\Vert {\textbf {Y}}_i,{\textbf {Y}}_j \Vert ^{\Psi }$ to interpret the difference of two sets of data (${\textbf {Y}}_i$, ${\textbf {Y}}_j$) for the set of simulations $\Psi$. In the literature, the stress-strain graph is usually used to compare the data ${\textbf {Y}}_{0}$ of the experiment $\psi$ and the data ${\textbf {Y}}_*$ of the experiment simulation. To describe this difference in the article, we use the norm $\Vert {\textbf {Y}}_{0},{\textbf {Y}}_* \Vert ^{\psi }$ (see Section 2.2). The value of the norm can be interpreted as a measure of the quality or accuracy of the solution. The norm can be defined in different ways, which implies different values. Therefore, we present a comparison of the curves in the stress-strain graph with the norm value for one experiment. Figure 2 shows the relationship between the difference of the curves and the norm value for the reduced axisymmetric model (see Section 3.1). The dependence is shown for the experiment $\psi = 1$ with parameters $P_0$, see Table 2.

From Figure 2 it can be seen that the value of the norm $\Vert {\textbf {Y}}_{0},{\textbf {Y}}_1 \Vert ^{\psi } = 0.01$ represents the curves that differ significantly over almost the entire length of the curve and are not acceptable. The norm value $\Vert {\textbf {Y}}_{0},{\textbf {Y}}_2 \Vert ^{\psi } = 0.004$ represents the curves that are still quite different, especially in the initial part of the curve. At least, the norm value $\Vert {\textbf {Y}}_{0},{\textbf {Y}}_2 \Vert ^{\psi } = 0.001$ represents the curves that almost overlap. Based on the figure, norm values equal to (0.001) or less (0.0005, 0.00025) were chosen for further tests. The norm values (less than 0.001) represent a good agreement of the curves for the tension tests. We will assume that similar values of the norm are also sufficient for a set of experiments ($\Vert {\textbf {Y}}_{0},{\textbf {Y}}_* \Vert ^{\Psi }$).

Note: For real experiments (data), this value of the norm may not be achievable. Therefore, for real data, results with a norm value greater than 0.001 can also be presented.

### Analysis of local dependence

The analysis includes three subsequent steps. The first step is based on the neighborhood definitions in Section 2 for an output data set and parameters. Unifies changes in parameter values so that they are defined by only one value $\epsilon$. In the second step, the local dependence matrix is solved. In the last step, the coefficients of the local dependence are solved.

For the given value ${\textbf {P}}_0$ (Table 2) and $\epsilon = 0.0005$, the values ${\textbf {P}}^{i}$, that is, $\varepsilon _i: i = 1, 2 \cdots N$ were found ($\Vert {\textbf {Y}}_0,{\textbf {Y}}_{i} \Vert ^\Psi \approx \epsilon$). It can describe symbolically as follows:

25 $$\begin{aligned} (\Vert {\textbf {Y}}_0,{\textbf {Y}}_{i} \Vert ^\Psi \approx \epsilon \rightarrow \varepsilon _i): i = 1, 2, \cdots N. \end{aligned}$$

The resulting values $\varepsilon _i$ for 8 parameters are shown in the following Table 3.

**Table 3:** Estimations of $\varepsilon _i$ values for $\epsilon \approx 0.0005$.

i	1	2	3	4	5	6	7	8
$p_i$	$s_0$ [*MPa*]	*Q*/*R* [*K*]	*A* [1/*s*]	$\xi$ $[-]$	*m* $[-]$	$h_0$ [*MPa*]	$\hat{s}$ [*MPa*]	*n* $[-]$
$\epsilon$ [-]	0.000500	0.000499	0.000499	0.000499	0.000499	0.000500	0.000498	0.000499
$\varepsilon _i$ [-]	0.6217	53.33	1058.35	0.04	0.002458	997.66	0.343	0.000815

Since the local dependence is always based on two parameters, the last parameter is not needed for the next analysis. The values for the last parameter are as follows $p_9= a$, $\varepsilon _9 = 0.0833$, $\epsilon = 0.000501$. Since only one $\varepsilon _i$ value is always searched, which is sufficient to gradually increase, the algorithm used will not be described. The following criterion $\mid \Vert {\textbf {Y}}_0,{\textbf {Y}}_{i} \Vert ^\Psi - \epsilon \mid < 0.000002$ was used to terminate the search.

In the second step, we use nearly the same algorithm (Definition 3). ${\textbf {P}}^i$, i.e. $\varepsilon _i: i = 1, 2 \cdots (N-1)$, were used as reference values (Table 3). We consider the value of $\varepsilon _j$ as a variable and from the equation (20) we determine the values of structurally local dependence $\lambda _{i,j}$ for all parameters. The resulting matrix is as follows:

26 $$\begin{aligned} \Lambda = \begin{bmatrix} 0 & 7.61 & 7.63 & 7.60 & 7.72 & 8.44 & 9.28 & 9.39 & 8.85 \\ 7.61 & 0 & 0.716& 1.38 & 1.82 & 3.78 & 3.60 & 4.77 & 3.31 \\ 7.63 & 0.716 & 0 & 1.67 & 2.15 & 4.00 & 3.84 & 5.12 & 3.60 \\ 7.60 & 1.38 & 1.67 & 0 & 0.512 & 3.08 & 3.36 & 4.20 & 2.81 \\ 7.72 & 1.82 & 2.15 & 0.512 & 0 & 2.93 & 3.20 & 3.80 & 2.58 \\ 8.44 & 3.78 & 4.00 & 3.08 & 2.93 & 0 & 3.79 & 4.22 & 1.65 \\ 9.28 & 3.60 & 3.84 & 3.36 & 3.20 & 3.79 & 0 & 2.13 & 2.27 \\ 9.39 & 4.77 & 5.12 & 4.20 & 3.80 & 4.22 & 2.13 & 0 & 3.17 \\ 8.85 & 3.31 & 3.60 & 2.81 & 2.58 & 1.65 & 2.27 & 3.17 & 0\\ \end{bmatrix}10^{-4} \end{aligned}$$

We calculate half of the matrix; we assume a symmetrical variant, where the other half is only supplemented. For the solution, the same algorithm was used as in the previous step. The search was terminated when the difference of two consecutive steps was less than 0.000002.

In the last step, the local dependence coefficients (22) are calculated from the matrix $\Lambda$. Table 4 shows the first 4 steps, when we first solve the underlined parameter. This one has the least value, and we assume will be the easiest to replace. If it is dependent, we continue in the table with the second row, where the values of local dependence coefficients are recalculated but without the dependent parameter.

**Table 4:** The first four cycles of the local dependence for global testing of structural dependence.

i	1	2	3	4	5	6	7	8	9
$p_i$	$s_0$ [*MPa*]	*Q*/*R* [*K*]	*A* [1/*s*]	$\xi$ $[-]$	*m* $[-]$	$h_0$ [*MPa*]	$\hat{s}$ [*MPa*]	*n* $[-]$	*a* $[-]$
Step 1,$\Lambda _i$	0.0067	0.0027	0.0029	0.00246	0.00247	0.0032	0.0032	0.0037	0.0028
Step 2, $\Lambda _i$	0.0059	0.0026	0.0027	-	0.0024	0.0029	0.0028	0.0033	0.0025
Step 3, $\Lambda _i$	0.0051	0.0024	0.0025	-	-	0.0026	0.0026	0.0029	0.0023
Step 4, $\Lambda _i$	0.0042	0.00205	0.0021	-	-	0.0024	0.0023	0.0026	-

The smallest agreement is with the parameter $i=1$ ($s_0$), which has almost twice the value of the second parameter on the order $i=8$ (*n*). On the other hand, the best match is for the parameter $i = 4$ ($\xi$), and this parameter will be tested first. The table shows the first 4 steps with the extraction parameters $\xi$, *m*, *a*, *Q*/*R*.

### Analysis of global structural dependence

The following sections describe the results of individual test cycles. The results of the solution are presented in a table, where the first row contains the values of the fictive parameters ${\textbf {P}}_0$ ($\Vert {\textbf {Y}}_{0},{\textbf {Y}}_0 \Vert ^\Psi = 0$). In the next rows, the solutions are sorted according to the size of the value of the parameter tested. The tested parameters are selected from the global intervals and the identified parameters must belong only to the basic intervals (see Section 3.2). The last column contains the value $\Vert {\textbf {Y}}_{0},{\textbf {Y}}_i \Vert ^\Psi$.

The calculation proceeds in a cycle; the parameter selected in Section 3.5, Table 4 as the most easily replaceable is tested locally for the condition (27). The parameter was tested in the selected range (Section 3.2), the table shows 9 selected parameter points.

27 $$\begin{aligned} \Vert {\textbf {Y}}_{0},{\textbf {Y}}_{i} \Vert ^\Psi \le \epsilon : i = 1..10. \end{aligned}$$

If the condition is met for all parameter points, the parameter is replaced by a constant in the appropriate range. The parameter value does not change further. The cycle continues by selecting and testing the next parameter. Otherwise, the condition is not met in a significant part of the interval; then, the parameter is independent, and the analysis is terminated. The FEMU approach (see Section 2.6).

The quality of the result is determined by the number of checked points (we choose 9 points) and the value of $\epsilon$, see (27). We choose the value according to Section 3.4, that is, $\epsilon = 0.001$. The goal was to find values for $\epsilon = 0.0005$. Variants above the value $\epsilon = 0.001$ were considered unsatisfactory.

**Parameter** $\xi$: From Table 4, the first parameter tested is $p_4 = \xi$ (see also^5^). The value of the parameter usually varies in the interval $\xi \in [1,10]$ (see Section 3.2). The results of the solution for the selected points are shown in Table 5 A.

**Table 5:** Parameters $${\textbf {P}}_i$$.

i	1	2	3	4	5	6	7	8	9	
$p_i$	$s_0$ [*MPa*]	*Q*/*R* [*K*]	*A* [1/*s*]	$\xi$ $[-]$	*m* $[-]$	$h_0$ [*MPa*]	$\hat{s}$ [*MPa*]	*n* $[-]$	*a* $[-]$	$\Vert {\textbf {Y}}_{0},{\textbf {Y}}_i \Vert ^\Psi$ $[-]$
A/ $\varepsilon = 0.0005$, $\xi \in [1,10]$										
0	21.40	10494	6103.5	4	0.2	6140	26.4	0.01	1.91	0
1	7.62	9899	9082.0	1	0.15	5086	11.18	0.0080	3.37	0.00050
2	14.97	9717	5640.1	2	0.15	7585	20.47	0.0079	2.63	0.00049
3	17.87	9968	4841.8	3	0.19	7218	23.41	0.0061	2.06	0.00050
4	21.40	10497	6103.7	5	0.26	6140	26.40	0.010	1.91	0.00050
5	23.79	11229	7325.9	6	0.25	6777	27.64	0.012	1.85	0.00048
6	24.65	11340	7241.6	7	0.28	6803	28.46	0.012	1.80	0.00049
7	26.34	11249	7510.3	8	0.31	7141	30.50	0.012	1.80	0.00050
8	27.67	11313	7366.1	9	0.33	7084	32.18	0.012	1.83	0.00050
9	27.49	11360	7412.7	10	0.37	6890	32.46	0.011	1.77	0.00050
B/ $\varepsilon = 0.001$ for $m \in [0.11,4]$										
1	39.52	9138	3358.3	4	0.11	10777	49.1	0.0114	1.74	0.00100
2	34.36	9131	3201.3	4	0.14	10707	41.8	0.0100	1.74	0.00091
3	30.62	9329	3024.5	4	0.16	9541	37.1	0.0117	2.07	0.00098
4	20.63	10283	6197.0	4	0.22	6151	25.5	0.0099	1.93	0.00049
5	14.18	10681	6367.0	4	0.32	5994	17.90	0.0087	2.31	0.00049
6	8.09	11364	7061.5	4	0.52	6927	11.03	0.0085	3.26	0.00050
7	4.33	11221	4139.5	4	1	4302	6.162	0.0065	3.40	0.00050
8	2.19	11477	4267.0	4	2	3908	3.085	0.0051	3.90	0.00095
9	1.55	10918	4442.8	4	3	3904	2.373	0.0046	4.32	0.00089
10	1.20	10879	4657.9	4	4	3960	1.840	0.0045	4.66	0.00093
C/ $\varepsilon = 0.001$ for $a \in [1.00001,2]$										
1	4.38	11111	2668.1	4	1	339.0	4.957	0.0095	1.00001	0.00049
2	4.38	11111	2668.1	4	1	339.0	4.957	0.0095	1.1	0.00082
3	4.26	11229	2835.2	4	1	501.7	4.851	0.0099	1.2	0.00049
4	4.26	11229	2835.2	4	1	501.7	4.851	0.0099	1.3	0.00079
5	4.16	11356	2920.3	4	1	643.5	4.777	0.0103	1.4	0.00048
6	4.16	11356	2920.3	4	1	643.5	4.777	0.0103	1.5	0.00078
7	4.19	11282	3021.9	4	1	991.4	4.991	0.0089	1.6	0.00049
8	4.19	11282	3021.9	4	1	991.4	4.991	0.0089	1.7	0.00072
9	4.44	10924	2752.9	4	1	1239.6	5.509	0.0077	1.8	0.00049
10	4.34	11023	2674.2	4	1	1720.7	5.547	0.0066	2	0.00049
D/ $\varepsilon = 0.001$ for $Q/R \in [6000,15000]$										
1	5.12	6000	0.00797	4	1	1435	5.85	0.0132	2	0.00267
2	5.42	7000	0.309	4	1	1329	7.46	0.0016	2	0.00223
3	4.70	8000	0.897	4	1	1459	5.37	0.0130	2	0.00136
4	4.50	9000	9.322	4	1	1428	5.23	0.0120	2	0.00078
5	4.48	10000	199.6	4	1	1863	5.57	0.0077	2	0.00049
6	4.47	11000	3399	4	1	1389	5.95	0.0047	2	0.00049
7	3.97	12000	9679	4	1	1281	4.85	0.0093	2	0.00069
8	3.82	13000	96413	4	1	1242	4.73	0.0086	2	0.0011
9	3.67	14000	934385	4	1	1204	4.61	0.0079	2	0.0015
10	3.13	15000	940916	4	1	1184	3.60	0.0112	2	0.0020

For all values tested for the parameter $\xi$, the condition (27) is met, and therefore the parameter $\xi$ is globally dependent. For the next analysis, the parameter $\xi$ is frozen on a value $\xi =4$. We shall further consider this parameter as a constant.

**Parameter** *m*: From Table 4, the second parameter tested is $p_5 = m$. The value of the parameter usually varies in the interval $m \in [0.1-4]$ (see Section 3.2). The results of the solution for the selected points are in Table 5 B. For all values tested for the parameter *m*, the condition (27) is met, and therefore the parameter *m* is globally dependent. For the next analysis, the parameter *m* is frozen on a value $m = 1$. We shall further consider this parameter as a constant.

**Parameter** *a*: From Table 4 is the third testing parameter $p_9 = a$. The value of the parameter usually varies in the interval $a \in [1.00001,2]$ (see Section 3.2). The results of the solution for the selected points are in Table 5 C. For all values tested of the parameter *a*, the condition (27) is met, and therefore the parameter *a* is global dependent. For the next analysis, the parameter is frozen *a* on a value $a=2$. We shall further consider this parameter as a known constant.

**Parametr** *Q*/*R*: From Table 4 is the fourth testing parameter $p_2 = Q/R$. The value of the parameter usually varies in the interval $a \in [6000,15000]$ (see Section 3.2). The results of the solution for the selected points are in Table 5 D. For all values tested for the parameter *Q*/*R*, the condition (27) is met only for the variants denoted as 4, 5, 6, 7, and therefore the parameter *Q*/*R* is globally independent. Due to the numerical solution used, it can be said that:

1. That the parameter *Q*/*R* is globally independent.
2. Or, the algorithm used cannot find a minimum even though it exists.

**Summary of analysis:** we assume that the parameters $s_0$, *Q*/*R*, *A*, $h_0$, $\hat{s}$, *n* are independent.

## Validation

In the previous sections, the parameters of the Anand model were divided into two groups: The independent parameters ($s_0$, *Q*/*R*, *A*, $h_0$, $\hat{s}$, *n*), and the dependent parameters ($\xi =4$, $m=1$, $a=2$), which were replaced using the selected constants.

The verification of results is achieved through different approaches, each of which is based on the following three questions: Do we get the same resulting parameters even with different initial values? What effect does the simulation model used have on the result? Will the behaviour be the same when using real experimental data?

These questions are elaborated in the following five subsections.

- The purpose of this phase is to determine whether the reduced set of parameters leads to the identification of their unique values (Section 4.1). A set of fictitious experiments that are not burdened by measurement error will be used for the solution. The uniqueness will be tested by varying the values of the input parameters.
- The purpose of this phase is to check whether the results obtained for simulations with one axisymmetric element give similar results for a larger number of solid elements of type SOLID186 (Section 4.2). A set of fictitious experiments that are not burdened by measurement error will be used for the solution.
- The purpose of this phase is to check whether the results obtained for tensile tests will be similar for compression tests (Section 4.3). These tests are also used to determine the values of the material parameters^9^. In compression tests, there is a more significant change in the shape of the sample during the experiment and, therefore, a change in the stress distribution. A set of fictitious experiments that are not burdened by measurement error will be used for the solution.
- The purpose of this phase is to determine whether the reduced set of parameters leads to the determination of their unique values (Section 4.4). A large set of real experiments will be used for the solution, which is burdened with a small measurement error. The uniqueness will be tested by varying the values of the input parameters.
- The purpose of this stage is to determine the values of the reduced set of parameters in a standard way (Section 4.5) and to check whether they will lead to an error value similar to that obtained by using the unreduced set of parameters. A small set of real experiments will be used for the solution, which is burdened with a measurement error.

### Effect of different initial values of parameters

The aim of this section is to verify whether different initial parameter values lead to the same result. A set of 25 fictitious experiments and the simplest possible simulation model are used. We assume that if the parameters ($s_0$, *Q*/*R*, *A*, $h_0$, $\hat{s}$, *n*) are independent, they should lead to the same resulting values (unambiguous) regardless of the initial parameter values used for identification (see (1)).

The set of 25 fictive experiments was used, the model and loading states are described in (Section 3.1). The fictive experiments were generated by parameters denoted as $i=0$ in Table 6 A, for values of frozen parameters ($\xi =4$, $m=0.2$, $a=1.9075$).

Initial sets of parameter values were selected from^11^. The frozen parameters were rewritten to the corresponding values ($\xi =4$, $m=1$, $a=2$). After this change, the parameter values no longer correspond to the published results. The initial parameter values for the eight variants are shown in Table 6 A. The variants are distinguished according to the index *i*.

**Table 6:** Sets of parameters - values during identification process.

i	1	2	3	6	7	8	
$p_i$	$s_0$ [*MPa*]	*Q*/*R* [*K*]	*A* [1/*s*]	$h_0$ [*MPa*]	$\hat{s}$ [*MPa*]	*n* $[-]$	$\Vert {\textbf {Y}}_{0},{\textbf {Y}}_i \Vert ^\Psi$ $[-]$
A/ Initial set of parameters - 1+8 variants.							
0, 1	21.398	10494.2	6103.5	6139.5	26.4	0.01	-
2	5.1	3468.0	0.000931	49000	16.4	0.078	-
3	1.067	10413.3	8.247e7	5023.9	20.30	0.03247	-
4	3.3	9883.0	15.77	1076.9	3.15	0.0035	-
5	7.72	14100	1630000	58700	11.99	0.0017	-
6	32.64	7619.0	107.65	9002	86.28	0.0046	-
7	31.09	8900.0	22300	3321	73.81	0.0018	-
8	2.3	9970.0	17.99	1525	2.536	0.028	-
B/ level $\epsilon \approx 0.001$							
1	4.50	10763.2	3993.5	2659.9	5.89	0.005449	0.0012
2	4.79	9962.7	364.9	2033.4	6.76	8.690e-07	0.001
3	4.25	12299.7	78685.9	1357.0	5.34	0.006681	0.0013
4	4.63	9025.7	18.0	1183.4	6.08	0.005511	0.001
5	4.59	12032.4	70741.1	1308.3	6.35	0.001591	0.00097
6	4.39	9884.5	162.1	2790.5	5.61	0.005056	0.00097
7	4.63	11211.8	9878.3	597.5	6.69	0.00289	0.001
8	3.58	10560.2	15.38	1146.9	3.12	0.02436	0.001
C/ level $\epsilon \approx 0.00025$							
1	4.35	10980.2	2660.9	1720.2	5.53	0.006534	0.00029
2*	4.81	10517.4	2486.2	1313.1	6.93	1.052e-07	0.00055
3	4.42	11174.2	4855.1	1227.3	5.75	0.006434	0.00022
4	3.91	10141.2	29.6	1298.5	4.35	0.0139	0.00025
5	4.65	10592	1881.2	1349.0	6.34	0.003283	0.00029
6	4.46	9876.2	100.0	1356.0	5.68	0.007261	0.00029
7	4.67	10531.3	1500.7	1141.1	6.38	0.003605	0.00026
8	3.80	10216.4	24.2	1536.3	3.95	0.01674	0.00022

For better data orientation, we describe the parameter values using the arithmetic mean and the corrected sample standard deviation. The initial values of the unfrozen parameters (Table 6 A) are described as follows:

- $$s_0 = 13.08 \pm 13.23$$
- $$h_0 = 16723.5 \pm 23200.2$$
- $$Q/r = 9355.9 \pm 3011.1$$
- $$\hat{s} = 30.1 \pm 32.0$$
- $$A = 10515443 \pm 29077574$$
- $$n = 0.02 \pm 0.026$$

From Figure 2 it can be stated that for the value of $\epsilon \approx 0.001$, visually almost identical curves can be obtained. The first identification was made at this level of precision and the results are shown in Table 6 B. The values can be summarized as follows:

- $$s_0 = 4.42 \pm 0.38$$
- $$h_0 = 1634.6 \pm 780$$
- $$Q/r = 10717.5 \pm 1100$$
- $$\hat{s} = 5.73 \pm 1.2$$
- $$A = 20482 \pm 33700$$
- $$n = 0.006442 \pm 0.0076$$
- $$\Vert {\textbf {Y}}_{F \Psi },{\textbf {Y}}_i \Vert ^\Psi \approx 0.0011 \pm 0.00012$$

The next cycle of identification was performed at the level $\epsilon \approx 0.00025$. The results are presented in Table 6 C. Variant $i=2$ (marked 2*) could not be adjusted to the required value ($\epsilon \approx 0.0005$), so it was discarded. We assume that this is due to the parameter $n = 1.052e-07$, which is the only one that is very different from the other variants. This variant was not included in subsequent calculations. The values for the parameters listed in Table 6 C can be summarized as follows:

- $$s_0 = 4.32 \pm 0.34$$
- $$h_0 = 1375.5 \pm 195$$
- $$Q/r = 10501 \pm 464$$
- $$\hat{s} = 5.43 \pm 0.94$$
- $$A = 1578.8 \pm 1780$$
- $$n = 0.008251 \pm 0.0051$$
- $$\Vert {\textbf {Y}}_{F \Psi },{\textbf {Y}}_i \Vert ^\Psi \approx 0.00026 \pm 3.2e-5$$

I think the parameter variance values are acceptable for the first four parameters. But the last two parameters have almost the same variance value as their mean, so we will try to continue the identification process (without the $i=2$ option).

The least identification step was performed at the level $\epsilon \approx 0.00005$. The results are presented in Table 7. The values for the parameters can be summarized as follows:

- $$s_0 = 4.31 \pm 0.16$$
- $$h_0 = 1363.1 \pm 107.4$$
- $$Q/r = 10461.4 \pm 260$$
- $$\hat{s} = 5.28 \pm 0.43$$
- $$A = 592.9 \pm 480$$
- $$n = 0.009284 \pm 0.0025$$
- $$\Vert {\textbf {Y}}_{F \Psi },{\textbf {Y}}_i \Vert ^\Psi \approx 5.0e-5 \pm 1.1e-5$$

**Table 7:** The resulting parameters for the tested variants for the level $\epsilon \approx 0.00005$.

i	1	2	3	6	7	8	
$p_i$	$s_0$ [*MPa*]	*Q*/*R* [*K*]	*A* [1/*s*]	$h_0$ [*MPa*]	$\hat{s}$ [*MPa*]	*n* $[-]$	$\Vert {\textbf {Y}}_{0},{\textbf {Y}}_i \Vert ^\Psi$ $[-]$
1	4.39	10733.6	1091.8	1249.4	5.53	0.007992	5.8e-05
3	4.44	10561.9	789.8	1378.6	5.68	0.006855	5.7e-05
4	4.03	10310.8	87.1	1408.5	4.56	0.01322	5.0e-05
5	4.44	10533.0	731.8	1421.4	5.66	0.006958	5.6e-05
6	4.36	10115.7	144.2	1200.9	5.22	0.01055	3.5e-05
7	4.37	10781.2	1204.3	1364.1	5.48	0.007985	6.2e-05
8	4.15	10193.4	98.6	1518.7	4.84	0.01143	3.6e-05

in this last identification cycle, the value of the parameter *A* is still unstabilized (it has almost the same variance as their mean value). There are several possible solutions; for example, we can identify the parameter *A* by another method and use the value to reduce the allowable interval of the parameter. Or, if we have an estimate of the value of parameter *A*, we can use it as a frozen parameter and activate another parameter. However, this option has not been tested.

### Tension test

**Figure 3:**
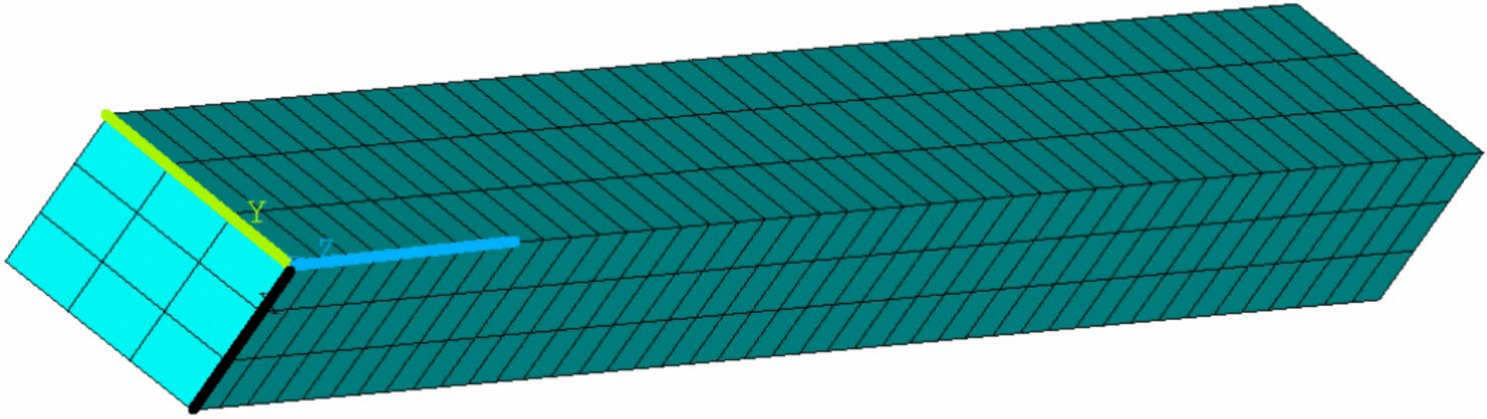
Mesh for simulation model of the tension test.

Previous tests were performed on a simplified model in one axisymmetric element (see Section 3.1). In this Section, standard specimen shapes for tension will be used. Different samples can be used for tensile tests. To create a regular mesh, we use the sample shape described in^43^. The simulation model includes a part of the sample with dimensions of 3mm $\times$ 4mm $\times$ 30mm. Only 1/4 of the sample is modeled, with dimensions of 1.5 mm $\times$ 2 mm $\times$ 30 mm. SOLID type elements with intermediate nodes are used. A regular mesh with brick-type elements (SOLID186) is used to simulate a tension test with an assumed uniform distribution of stress throughout the sample. The mesh is created from 387 elements (2448 nodes) of element type SOLID186 (3D 20-Node Structural Solid, intermediate nodes); see Figure 3.

The simulation model has two planes of symmetry; in Figure 3 the first plane is shown by blue / green lines and the second plane by blue / black lines. Displacements in the direction perpendicular to the given plane of symmetry are captured in the nodes in these planes. On the “left” side of the sample, the displacements of all nodes in the direction of the axis (shown in blue in the picture) are fixed. On the “right” side of the specimen, all nodes are assigned identical axial displacements (in the direction of the blue axis) corresponding to the test load. The displacement increases linearly with time, and its maximum value is 8.54 mm. Five different temperatures (20, 40, 60, 80, 100 $^{\circ }$C) and five different strain rates (multiply time coefficient 1, 0.1, 0.01, 0.001, 0.0001) are solved again. So, a total of 25 fictitious experiments.

Seven variants of the parameters listed in Table 7 are tested, and as an ideal solution we consider variant 0 again. We assume that if the influence is negligible, the norm value will be $\Vert {\textbf {Y}}_{F \Psi },{\textbf {Y}}_i \Vert ^\Psi \approx 0.00005$ for all variants tested.

The value of the norm $\Vert {\textbf {Y}},{\textbf {Y}}^k \Vert ^\Psi$ for the individual parameter variants is given in Table 8.

**Table 8:** The norm value for the tested variants of the parameter sets.

i	0	1	3	4	5	6	7	8
$\Vert {\textbf {Y}},{\textbf {Y}}^k \Vert ^\Psi$	0	5.8e-05	5.5e-05	2.7e-05	4.6e-05	7.7e-05	3.9e-05	4.1e-05

It can be seen from the results that values of the norm is at the expected level of 0.00005 (compare with Table 7). Therefore, the effect of using one axisymmetric element is low.

### Compression test

**Figure 4:**
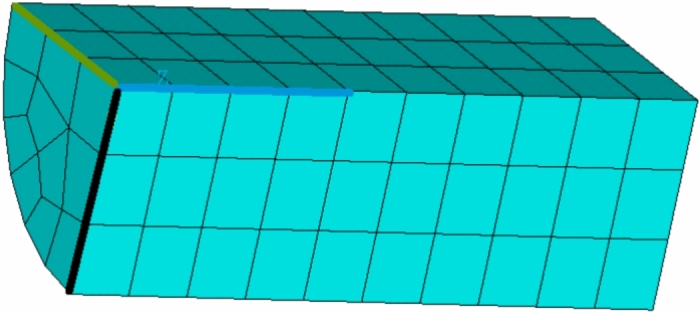
Mesh for simulation model of the compression test.

In this case, a compression test is simulated and a very regular mesh with brick-type elements is used. We used^9^ as a source for the compression test. The sample has the shape of a cylinder with a diameter of 12.7 mm and a length of 19.05 mm. The simulation model again includes only 1/4 of the sample. Its mesh consists of elements (110 elements, 686 nodes) of type SOLID186 (3D 20-Node Structural Solid, intermediate nodes), see Figure 4.

As with the tensile test (in the previous Section), symmetry is used, the planes of symmetry are marked with the same colours (blue/green, blue/black). On the “left” side of the sample, all nodes are fixed in all directions (blue, green, black axis). On the “right” side of the specimen, all nodes are assigned identical axial displacements (shown in blue) corresponding to the test load, and displacements perpendicular to this axis are fixed. The displacement decreased linearly with time and its minimum value is -8.54 mm. The total number of experiments is again 25 (Five different temperatures 20, 40, 60, 80, 100 $^{\circ }$C and five different strain rates, multiply time coefficient 1, 0.1, 0.01, 0.001, 0.0001).

Seven variants of the parameters listed in Table 7 are tested, and as fictive experiments we generate from variant 0 again. We assume that if the influence is negligible, the norm value will be $\Vert {\textbf {Y}}_{F \Psi },{\textbf {Y}}_i \Vert ^\Psi \approx 0.00005$ for all variants tested.

The value of the norm $\Vert {\textbf {Y}},{\textbf {Y}}^k \Vert ^\Psi$ for the individual parameter variants is given in Table 9.

**Table 9:** The norm value for the tested variants of the parameter sets.

k	0	1*	3	4	5	6*	7	8
$\Vert {\textbf {Y}},{\textbf {Y}}^k \Vert ^\Psi$	0	9.7e-05	7.7e-05	5.2e-05	7.3e-05	0.00021	4.2e-05	4.1e-05

**Figure 5:**
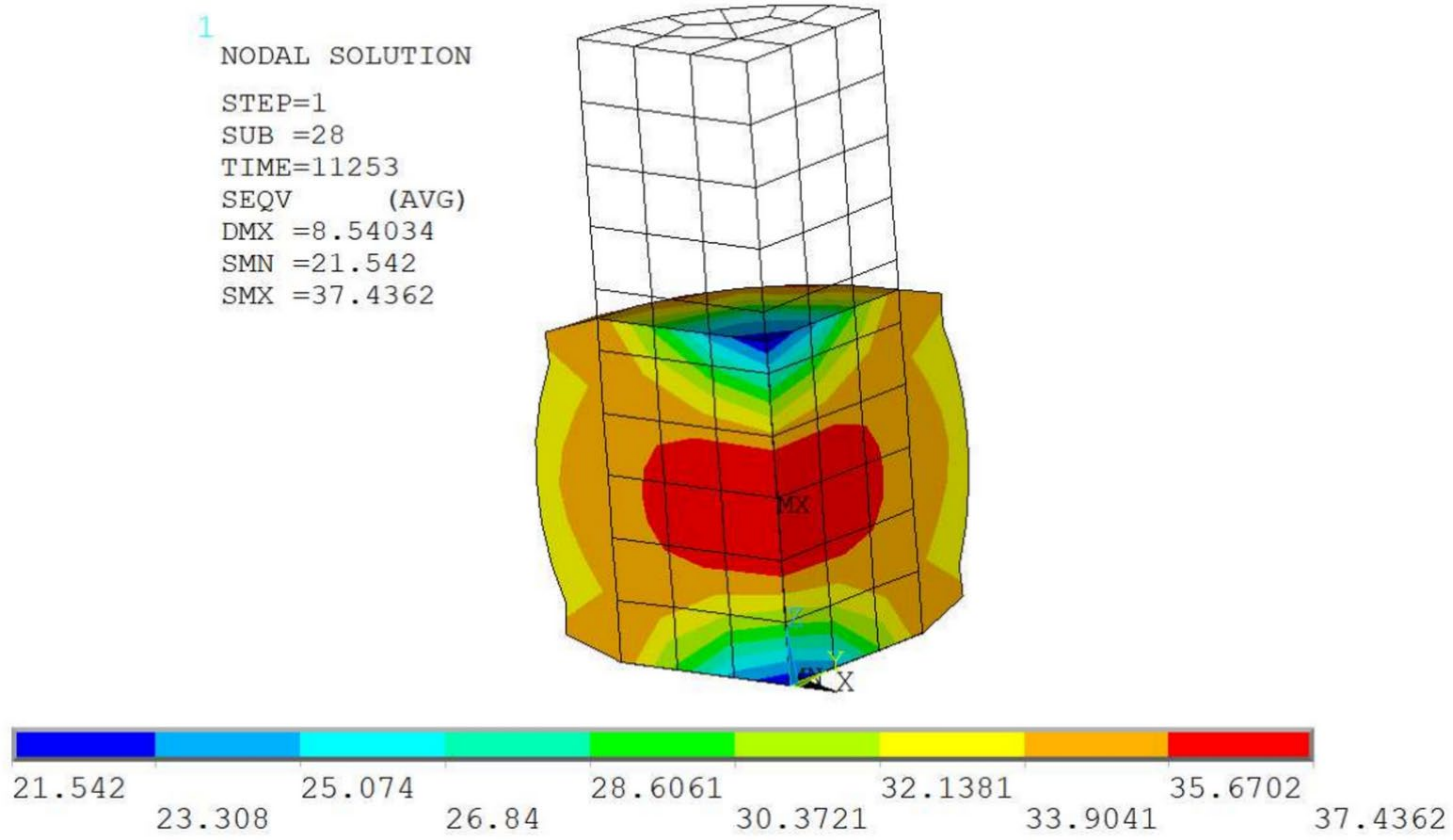
Reduced stress $[\text {MPa}]$ for maximum displacement of -8.54 mm and lowest loading rate.

It can be seen from the results that values of the norm is at the expected level of 0.00005. Variants 1, 6, marked with “*” gave worse results. Compared to the variant of the tensile test, an unevenly distributed stress field is in the sample, see Figure 5.

It is hypothesised that both types of experiments are interchangeable and lead to the same parameter values. Five results were within the acceptable range, while two were already around twice (or more) the required value. This raises the question whether a combination of compression and tensile tests, which give more or less identical results, could reduce the dispersion of parameter values. However, these hypotheses were not tested in this work.

### Validation on set of experiments 1

In this section, we try to validate (1) numerically. First, a set of initial parameter values was selected. The frozen parameter values were overwritten ($\xi$, *m*, *a*) with constants, as in the previous section. Since the published parameter values ($\xi$, *m*, *a*) differed for the calculated variants, the values of $\varepsilon$ also differed. Therefore, the set was unified at the value of $\varepsilon \approx 0.1$. The parameters for smaller values of $\varepsilon$ up to $\varepsilon \approx 0.001$ were searched. Parameters for lower values of $\varepsilon \approx 0.0009$ could not be found. The FEMU algorithm used is described in Section 2.6 (Nelder-Mead simplex method with adaptive parameters).

We use data from tension tests for the material SAC305 presented in^10^. The material SAC (alloy) is typical for Anand model.The paper presents a set of 15 curves with three strain rates and five temperatures. The data are in the form of strain-stress curves, and these were used for validation in this Section. The data includes a set of tensile experiments at 5 different temperatures ($25, 50, 75, 100 \text { and } 125 ^\circ C$) and 3 different strain rates ($.001, .0001 \text { and }.00001(1/s)$). For each set of test conditions, 10 samples were tested and the resulting curves were determined as the average of these experiments. For identification of material parameters, non-linear least squares were used in^10^.

The conclusions of the previous sections are verified by experimental data. When using real experiments, a larger scatter of data can be expected, so the value of ($\varepsilon \approx 0.1, \text { and then gradually } 0.033, 0.01, 0.0033, 0.001$) was chosen to be higher and gradually reduced during the solution. However, for the data used^10^, a solution was found for $\varepsilon = 0.001$.

The groups of parameters with different values were selected from^11^. The frozen parameters were rewritten to the corresponding constants ($\xi =4$, $m=1$, $a=2$), as was used in the previous section. After this change, the parameters no longer correspond to the published results. The initial parameter values for the eight variants are shown in Table 6 A.

From the experiments published in^10^, all tension tests with 5 different temperatures ($25^{\circ }$C, $50^{\circ }$C, $75^{\circ }$C, $100^{\circ }$C, $125^{\circ }$C) and 3 different deformation rates (0.001, 0.0001, 0.00001) were used. A total of 15 curves were used, each varying in temperature or deformation rate. The test sample has the form of a tape with dimensions of 80 x 3 x 0.5 mm.

**Figure 6:**
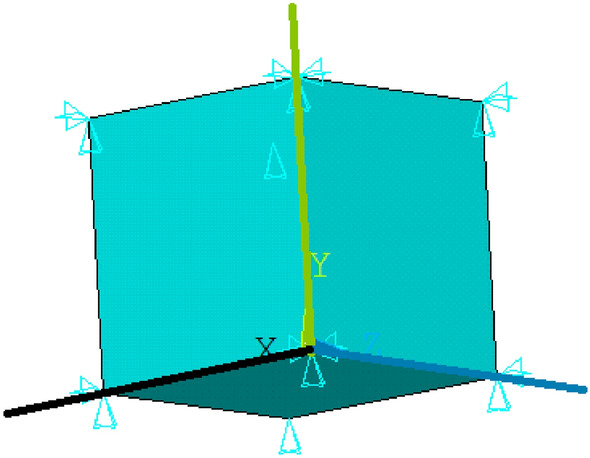
One element as the simulation model of the experiment set 1.

To simplify the calibration process, a single brick-type element (8 nodes) was used as the FE model; see Figure 6.

The dimensions of the model are 1 x 1 x 1mm, when considering the initial dimensions, the strain corresponds to the displacement and the stress corresponds to the force in the nodes. The model includes three planes of symmetry in which displacements in the direction perpendicular to the given plane of symmetry are fixed. The upper surface (corner nodes) is then moved in the vertical direction at the required speed (according to the given strain rates).

Tuning is performed starting from a set of initial values of the parameter vectors. The initial values of the parameter vectors that are used are listed in Table 6. The tuning is always completed when a certain norm value is reached, and the following five values are used ($\epsilon \approx 0.1, \text { and then gradually } 0.033, 0.01, 0.0033, 0.001$). A basic description of the model and experiments is given in Section 4.2. The frozen parameters $\xi = 4$, $m=1$, and $a = 2$ were used to calibrate the reduced set of parameters. In^10^ $\xi = 4$, $m=0.25$, and $a = 1.78$ were presented.

The first tuning cycle unified the values of $\varepsilon \approx 0.1$. Results with this value are not usable but show the initial variance of the parameters. The vector of parameters are shown in Table 10 A, and it can be summarized as follows:

- $$s_0 = 5.47 \pm 4.4$$
- $$Q/r = 9580 \pm 3118$$
- $$A = 16958544 \pm 44231171$$
- $$h_0 = 16253 \pm 22839$$
- $$\hat{s} = 11.06 \pm 5.32$$
- $$n = 0.0148 \pm 0.0160$$
- $$\Vert {\textbf {Y}}_{0},{\textbf {Y}}_i \Vert ^\Psi = 0.101 \pm 0.006$$

The first part of the table shows the parameters taken from the literature^10^.

In the next three tuning cycles, the value of $\varepsilon$ gradually decreased from 0.033 to 0.0033. The values of the resulting parameters are shown in Table 10 B, C, D.

**Table 10:** Real experiment set No. 2, with $\varepsilon \approx 0.1, 0.033, 0.01, 0.0033, 0.001$.

i	1	2	3	6	7	8	
$p_i$	$s_0$ [*MPa*]	*Q*/*R* [*K*]	*A* [1/*s*]	$h_0$ [*MPa*]	$\hat{s}$ [*MPa*]	*n* $[-]$	$\Vert {\textbf {Y}}_{0},{\textbf {Y}}_i \Vert ^\Psi$ $[-]$
^10^ *	21	9320	3501	180000	30.2	0.01	0.00863
A/ $\epsilon = 0.1$							
1	14.75	8765.8	8934.64	6157.63	15.807	0.01024	0.1025
2	5.95	2730.2	0.001225	48845.59	11.635	0.05171	0.108
3	1.539	12956.3	133974623	5539.0	10.845	0.01285	0.108
4	3.386	10929.0	15.302	1066.6	3.181	0.003565	0.101
5	7.91	12006.0	1645743.3	61741.8	10.915	0.001820	0.088
6	7.543	7074.6	137.5	3832.1	18.234	0.006682	0.101
7	0.332	11448.0	38880.1	1295.4	15.284	0.003256	0.1
8	2.33	10728.8	18.017	1543.5	2.578	0.02813	0.1
B/ $\epsilon \approx 0.03$							
1	10.41	9734.5	10435.8	6742.89	8.4427	0.008465	0.0302
2	6.9	3907.4	0.0011498	52325.9	7.5977	0.03147	0.0321
3	1.707	14792.4	132119699	5703.1	5.8975	0.01254	0.0319
4	3.539	12505.3	14.656	1033.4	3.1097	0.003533	0.0297
5	7.95	11817.4	1657437.7	62274.6	9.7159	0.001935	0.0308
6	8.106	7816.8	126.36	4004.5	14.6946	0.006647	0.0299
7	0.434	16826.8	28660.3	2258.0	3.6070	0.003169	0.031
8	2.398	11643.5	17.89	1556.3	2.6267	0.02807	0.0321
C/ $\epsilon \approx 0.01$							
1	6.238	10649.6	10506.8	7097.3	8.218	0.008778	0.00983
2	3.76	6687.5	0.001068	49739.2	4.056	0.01974	0.0097
3	1.825	17710.8	121734678	6427.9	3.4455	0.01141	0.00983
4	5.93	9884.0	19.186	512.48	4.4745	0.004974	0.0097
5	6.94	12903.7	1522785.	59800.5	6.9339	0.002136	0.0091
6	8.513	8626.5	132.572	3589.8	9.6274	0.006858	0.00984
7	0.47	12909.5	2650.1	12082.5	4.7384	0.002860	0.00986
8	2.398	12358.5	17.921	1545.62	2.6660	0.02821	0.0093
D/ $\epsilon \approx 0.0033$							
1	6.241	11534.5	10565.5	7429.62	6.173	0.007625	0.0032
2	3.65	8574.9	0.001168	50342.7	1.793	0.03933	0.00302
3	3.929	16100	5806245	7682.36	3.843	0.008918	0.00329
4	6.392	9418.9	95.677	267.51	12.847	0.006344	0.003324
5	4.126	14538.1	1938632	68021.7	5.148	0.002084	0.00326
6	6.657	9567.2	129.897	3964.44	6.953	0.007085	0.00321
7	0.0699	10570.3	295.273	46859.1	5.804	0.005428	0.00328
8	2.97	12890.9	18.307	1552.16	2.264	0.02804	0.00303
E/ $\epsilon \approx 0.001$							
1	4.21	11402.1	65.82	5658.5	3.31	0.02	0.001
3	4.224	11550.2	80.6	3670.5	3.16	0.02688	0.001
4	4.349	11205.6	58.62	3475.4	3.38	0.02656	0.001
6	4.118	11204.7	17.58	4900.6	2.723	0.03103	0.001
8	4.0946	11120.8	13.39	2394.1	2.59	0.03601	0.001
2^1^	1.663	10721.6	0.00131	46555.9	1.1795	0.03762	0.001714
5^1^	3.960	13216.4	145308	61839.6	5.4853	0.003022	0.002588
7^1^	0.08797	10888.0	739.41	65646.8	5.7195	0.006348	0.00261

^1^ The variants for which a solution of the required quality were not found

The values for the parameters listed in Table 10 D ($\varepsilon \approx 0.0033$) can be summarized as follows:

- $$s_0 = 4.25 \pm 2.05$$
- $$Q/r = 11649.3 \pm 2490.8$$
- $$A = 969497.7 \pm 1934925.1$$
- $$h_0 = 23264.9 \pm 25395.5$$
- $$\hat{s} = 5.603 \pm 3.233$$
- $$n = 0.0131 \pm 0.0123$$
- $$\Vert {\textbf {Y}}_{0},{\textbf {Y}}_i \Vert ^\Psi = 0.0032 \pm 0.00011$$

Compared to the previous tuning cycles, the value of $\Vert {\textbf {Y}}_{0},{\textbf {Y}}_i \Vert ^\Psi$ has been reduced by almost two orders of magnitude ($0.1 - 0.0033$). Parameter values (0.0033) could already be used in practice (see Figure 2). However, the variance values have improved only slightly compared to the previous tuning cycles. This indicates that the value $\Vert {\textbf {Y}}_{F \Psi },{\textbf {Y}}_i \Vert ^\Psi$ is still too large.

The following turning cycle was performed for the norm value of 0.001. No solution could be found for all variants. The resulting values of the parameters are given in Table 11 E. The first part of the table shows the parameters for which a solution was found at the norm value 0.001, and the second part of the table shows the variants for which no solution of the required quality could be found. The values of the parameters listed in Table 10 E that meet the chosen accuracy criterion can be summarized as follows:

- $$s_0 = 4.198 \pm 0.09$$
- $$Q/r = 11296.68 \pm 156.88$$
- $$A = 47.20 \pm 26.9$$
- $$h_0 = 4019.81 \pm 1142.1$$
- $$\hat{s} = 3.0318 \pm 0.3175$$
- $$n = 0.0289 \pm 0.0041$$
- $$\Vert {\textbf {Y}}_{0},{\textbf {Y}}_i \Vert ^\Psi =0.001 \pm 4 \cdot 10^-7$$

Here we can see a significant decrease in the value of the variance compared to the previous turning cycle (0.0033). Based on these values, we can say that we were able to unambiguously determine the values of the parameters $s_0$, *Q*/*r*, $\hat{s}$ and *n*. The values of the parameters *A* and $h_0$ still show a rather large scatter, but were determined at least in order of magnitude ($A \approx 2000-6000$, $h_0 \approx 10-80$).

### Validation on set of experiments 2

The objective of this section was to obtain the best possible solution regardless of the number of cycles (the number of cycles usually is hundreds). The frozen parameters $\xi = 4$, $m=1$, and $a = 2$ were used to calibrate the reduced set of parameters. A value of at least $\epsilon \approx 0.001$ was chosen. Data published in^40^ were used.

**Figure 7:**
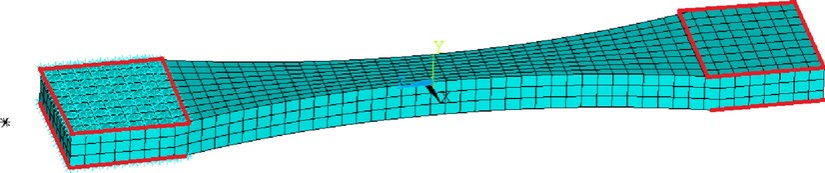
Mesh for simulation model of the experiment set 2.

Experiment set 2, as published in^40^, used samples made of ABS-M30 material and the Anand material model to simulate their behaviour. The identification of material parameters was performed using the FEMU approach and a genetic algorithm. The shape and dimensions of the sample were also detailed in the paper. This data was reanalyzed in^17^ using the same Anand material model and FEMU approach, but with the Nelder-Mead algorithm. The shape of the specimen is less typical (with a slight notch) and the material (ABS-M30) is also less typical for the Anand model. For clarity, the entire sample is presented here without symmetry; see Figure 7.

The data of the experiments were represented by the time / force curves for given deformation rates and temperatures. The boundary conditions are as follows: the right side of the sample is fixed (denoted by the red line in the figure) and the left side is loaded. On the loaded side of the specimen, all nodes are connected (using the MPC184 element type) to a single node. The loading of this node is defined by the displacement (deformation rate). From the experiments published in^40^, only tension tests with four different temperatures and three different deformation rates were used as follows:

- (1), Temperature $23 ^{\circ }$C, deformation rate 1.667 mm/s.
- (3), temperature $44 ^{\circ }$C, deformation rate 0.017 mm/s.
- (5), temperature $44 ^{\circ }$C, deformation rate 0.167 mm/s.
- (7), temperature $60 ^{\circ }$C, deformation rate 0.017 mm/s.
- (9), temperature $60 ^{\circ }$C, deformation rate 0.167 mm/s.
- (10), temperature $60 ^{\circ }$C, deformation rate 1.667 mm/s.
- (11), temperature $80 ^{\circ }$C, deformation rate 0.017 mm/s.

A total of 7 experiments were used; the number in parentheses indicates the order of the experiment in^40^.

To calibrate the parameters, approximately 5000 simulations/cycles were used. In Table 11, the following vectors of parameters are presented: the values of^40^, the initial values, and the final values (all with the corresponding values of the objective function). The frozen parameters in^40^ have the values $\xi = 5.82$, $m=0.213$, and $a = 3.527$, while the values $\xi = 4$, $m=1$, and $a = 2$ were used for calibration in this study.

**Table 11:** Real experiment set No. 2.

i	1	2	3	6	7	8	
$p_i$	$s_0$ [*MPa*]	*Q*/*R* [*K*]	*A* [1/*s*]	$h_0$ [*MPa*]	$\hat{s}$ [*MPa*]	*n* $[-]$	$\Vert {\textbf {Y}}_{F \Psi },{\textbf {Y}}_i \Vert ^\Psi$
^40^ *	18	9486	3263	138175	43.6	0.0226	0.00616
0	15	5000	107.79	1725.0	26.4	0.01	0.137
$\sim$ 5000	7.21	9883.5	59312	348.5	9.968	0.000154	0.0038

Data from Table 11 show that the reduced set of parameters of the Anand model is sufficient to identify the parameter values. Compared to^40^, a higher quality solution was achieved; therefore, even if a solution of the required quality was not found ($\epsilon \approx 0.001$), we consider this step satisfactory.

## Discussion

The proposed procedure to determine the structural dependence of a parameter, described in Section 2.4, is based on a numerical approach. At least a few alternatives to this procedure can be found, for example:

- Analysis of Anand model directly,
- or exploiting the physical behaviour of the parameters.

For example, in^5^, based on the analysis of Anand model, the value of the parameter $\xi$ is “frozen” at the value 7. In this article, we reached a similar conclusion using a different procedure; in Section 3.5, Table 4, the parameter $\xi$ is determined to be the easiest to replace by the other parameters. In^5^ a normalised sensitivity matrix (simplified as $\frac{\partial \Vert {\textbf {Y}}_{0},{\textbf {Y}}_i \Vert ^\Psi }{\partial p_i}$) is used, but the paper also differs from our conclusions. For example, in^5^ Parameter $s_0$, $C = A exp\left( -\frac{Q}{RT}\right)$ has the least effect on the cost function. In our study, the parameter $s_0$ is the most difficult to replace with the other parameters (Section 3.5, Table 4), and the parameters *Q*/*R* and *A* are the fourth and fifth easiest to replace, respectively. The sensitivity matrix is also reflected in the results; the parameters *A* and $h_0$ have the largest variance (see previous section). The discrepancy in the parameter $s_0$ is explained by the fact that although the parameter has a low sensitivity, its influence on the observed curves is different enough from the other parameters to be tuned.

**Figure 8:**
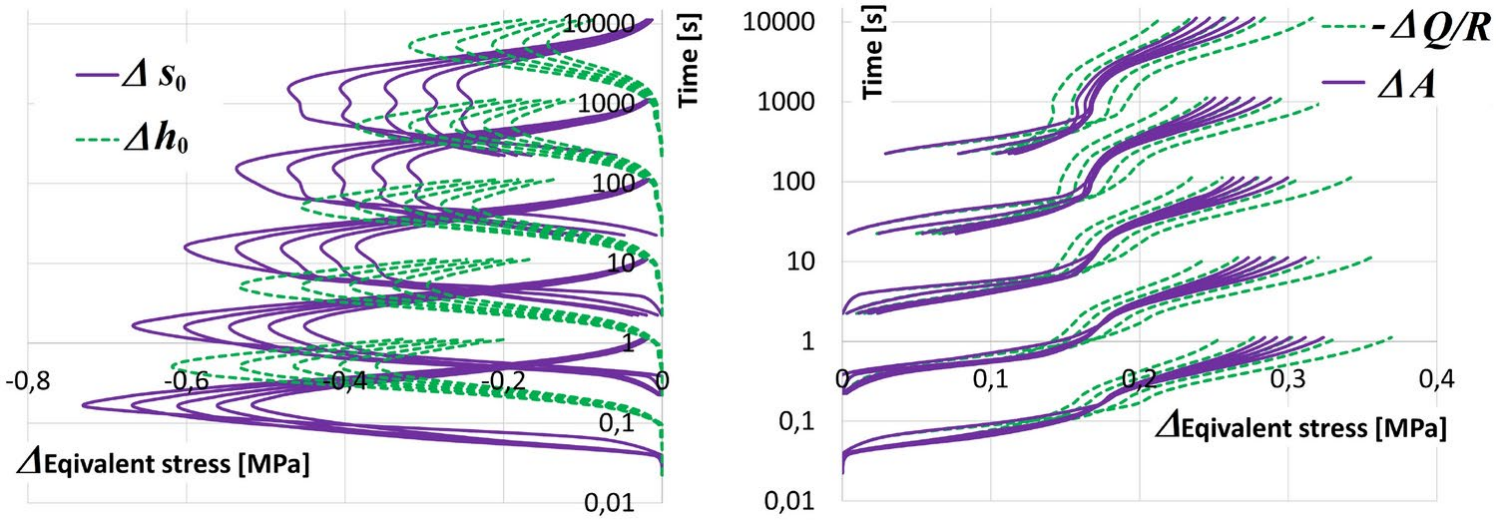
Tree graph for parameters $s_0$, $h_0$, *Q*/*R*, *A*.

**Figure 9:**
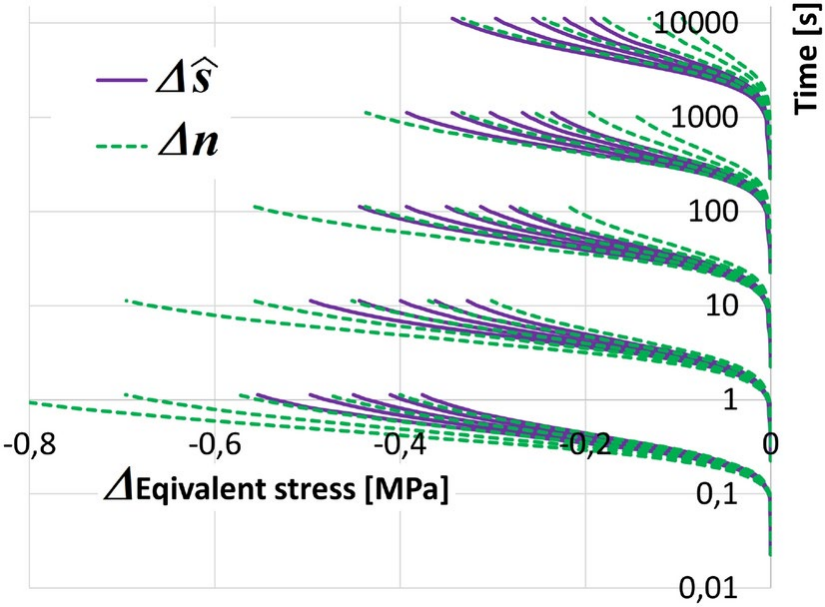
Tree graph for parameters $\hat{s}$, *n*.

An alternative may be a physical approach in which we assign certain properties to individual parameters. The influence of individual parameters on the resulting curves is not typical for Anand model. We tested this behaviour for the “independent” parameters found $s_0$, *Q*/*R*, *A*, $h_0$, $\hat{s}$, *n*. A change in the value of one parameter will result in a change in the given set of curves; we plot this change. The changes in curves are represented by $\Delta \text {Equivalent stress}$, which is the difference between the initial curves (Table 2) and the final curves (change value of one selected parameter). In the graphs, they are labelled $\Delta s_0$, $\Delta Q/R$, $\Delta A$, $\Delta h_0$, $\Delta \hat{s}$, and $\Delta n$. All curves are divided into three graphs according to their shape. We use the parameter values defined in Section 3.3, Table 2 (${\textbf {P}}_0$). Changes in parameters are defined by the value $\varepsilon \approx 0.0005$, see Table 3. The resulting curves are shown in Figure 8 and Figure 9.

The following conclusions can be drawn from these figures:

- The parameters $s_0$ and $h_0$ create a wave, the shape is different from all other parameters, the peak of the wave is shifted in both curves. We can assume that both parameters have an unmistakable influence.
- The influence of parameters $\hat{s}$ and *n* increases over time. They are very similar in shape, but their distribution on the timeline is different. Their combination will significantly influence the behaviour at different rates of deformation.
- The influence of parameters *Q*/*R*, *A* also increases with time, but the shape is significantly different compared to parameters $\hat{s}$, *n*. The curves for *Q*/*R*, *A* are very similar in shape, but a significant difference can be seen at different temperatures. Their combination will significantly influence the behaviour at different temperatures.

From this it can be concluded that all six parameters are needed to properly tune the material model. The material model is non-linear, so the behaviour for other ${\textbf {P}}$ values may differ.

This article uses fictitious experiments, where the data corresponding to the experiments are generated from the parameter values denoted as ${\textbf {P}}_{0}$. The primary benefit, as in the article stated, is that we can be certain that there are parameters that precisely correspond to the data; the data are not affected by measurement errors. Using fictitious experiments, we were able to achieve a norm value of 0.00005. Using experiments from the literature, we were able to achieve TOF value of 0.001.

In the first part of our study, we used only single-element models; this approach was used due to computational complexity. In^5^ it is also used as the only element in the preliminary study. In Section 4 the effect of the elements was tested and the compression test. In most of the tested cases, the assumptions were confirmed. However, in the compression test, two variants showed worse agreement compared to the tension with one element. It is therefore proposed to combine both tension and compression experiments for identification, which could reduce the dispersion of the resulting parameter values. However, due to the significant change in shape (for ductile materials), it is not possible to use only one element as a model. Which leads to an order of magnitude increase in computational times. Therefore, the use of the methodology presented in this article is not entirely appropriate in our opinion.

Parameters in material models often express the real behaviour observed in experiments. This is usually also expressed in the names of the individual parameters. For example: The Anand material model in several parts contains the expression $exp\left( \frac{Q}{RT} \right)$, which is based on the Arrhenius relation. The parameter *Q* is the activation energy, its value is related to a creep mechanism^44^. The creep mechanisms are determined by the temperature, and at a given temperature, several creep mechanisms can have an effect. In our paper there are parameters *Q*/*R*, and *A*. Knowledge of the values of these parameters could be used to determine the values of the frozen parameters. In the article, we did not deal with their optimal choice, for example:

- Is it possible to reduce the variance of *A* and $h_0$ by an appropriate choice of the values of the frozen parameters (*m*, $\xi$, *a*)?
- Is it possible to improve creep behaviour by appropriate choice of frozen parameter values (*m*, $\xi$, *a*)? The aim could be to identify parameters common to both types of tests typically used for Anand model (creep and tension tests).

## Conclusion

The article proposes a numerical procedure for determining the structural identifiability of parameters in non-linear physical models. The procedure is applied to the Anand model (Finite Element Method) which simulates the behaviour of the material during several experiments. For a set of tensile tests, it is shown that the number of parameters of the Anand model can be reduced from 9 to 6. Parameters that have been shown to have a negligible effect on the result are called frozen parameters. The frozen parameters found are *m*, $\xi$, *a*. During the tests, it was shown that decreasing TOF leads to a reduction in the variance of the parameter values. Thus, for a given set of tensile experiments, unique parameter values of Anand model can be found with a high degree of probability. This conclusion was established and validated on simple models, the set of tensile and compression tests. In the next work, we focus on the following questions: What is the impact of more complex simulation models (notched samples) and different types of experiments (e.g. creep tests)? How best to choose the values of the frozen parameters in Anand model? Another question is whether any of the procedures presented in the article could also be used for other material models. For example, for estimation of the number of back stress parts for Chaboche kinematic hardening model^45^ of multiaxial loading states, or to test the effects of a material model for combined material models.

## Supplementary information

The necessary information for the replication of the results is present in the manuscript. The interested reader may contact the corresponding author for further implementation details.

## Data Availability

Data is provided within the manuscript.
